# The Certification of Standard Reference Material 1979: Powder Diffraction Line Profile Standard for Crystallite Size Analysis

**DOI:** 10.6028/jres.125.020

**Published:** 2020-07-31

**Authors:** James P. Cline, Marcus H. Mendenhall, Joseph J. Ritter, David Black, Albert Henins, John E. Bonevich, Pamela S. Whitfield, James J. Filliben

**Affiliations:** 1 National Institute of Standards and Technology, Gaithersburg, MD 20899 USA; 3 Excelsus Structural Solutions, Villigen 5234, Switzerland; * Deceased

**Keywords:** crystallite size, line profile analysis, Standard Reference Material

## Abstract

This rather long-standing project has resulted in a National Institute of Standards and Technology (NIST) Standard Reference Material (SRM) for the analysis of crystallite size from a consideration of powder diffraction line profile broadening. It consists of two zinc oxide powders, one with a crystallite size distribution centered at approximately 15 nm, and a second centered at about 60 nm. These materials display the effects of stacking faults that broaden specific *hkl* reflections and a slight amount of microstrain broadening. Certification data were collected on the high-resolution powder diffractometer located at beamline 11-BM of the Advanced Photon Source, and on a NIST-built laboratory diffractometer equipped with a Johansson incident beam monochromator and position sensitive detector. Fourier transforms were extracted from the raw data using a modified, two-step profile fitting procedure that addressed the issue of accurate background determination. The mean column lengths, 〈*L*〉_area_ and 〈*L*〉_vol_, were then computed from the Fourier transforms of the specimen contribution for each reflection. Data were also analyzed with fundamental parameters approach refinements using broadening models to yield 〈*L*〉_area_ and 〈*L*〉_vol_ values. These values were consistent with the model-independent Fourier transform results; however, small discrepancies were noted for the 〈*L*〉_area_ values from both machines and both crystallite size ranges. The fundamental parameters approach fits to the laboratory data yielded the certified lattice parameters.

**Table 1:** List of all acronyms used in this work, and commonly used mathematical symbols.

APS	Advanced Photon Source	MCL	Mean Column Lengths
CoA	Certificate of Analysis	NLLSQ	Non-Linear Least-Squares
DBD	NIST Divergent Beam Diffractometer	PSD	Position Sensitive Detector
esd	estimated standard deviation	PSF	Profile Shape Function
FPA	Fundamental Parameters Approach	SI	International System of Units
FPAPC	Fundamental Parameters Approach Python Code	SRM	Standard Reference Material
FWHM	Full Width at Half Maximum	TEM	Transmission Electron Microscopy
GoF	Goodness of Fit	WH	Williamson-Hall
HCP	Hexagonal Close Packed	WPPM	Whole Powder Pattern Modeling
IBM	Incident Beam Monochromator	*ℱ*(ƒ)	Fourier transform of function ƒ
IPF	Instrument Profile Function	〈*L*〉_area_	Area-weighted mean column length
ISO	International Standards Organization	〈*L*〉_vol_	Volume-weighted mean column length
JCGM	Joint Committee for Guides in Metrology	⊗	Convolution operator for two functions
LPA	Line Profile Analysis	*d* *	λ/sin*θ*, the inverse *d*-spacing

## 1 General Introduction

The ability to use diffraction line profile shape to characterize the crystallite size of nano-scale materials is one of the many attributes of modern powder diffraction. The use of line profile analysis (LPA) has been extensive, as has been the research effort into the interpretation of results obtained from various strategies employed in its use. One way to assess performance of a complete measurement method is to acquire and analyze data from a standard sample with known properties. Toward this end the National Institute of Standards and Technology (NIST) has developed Standard Reference Material (SRM) 1979, a line-shape standard suitable for crystallite size calibrations. The certified values for the profile breadth of SRM 1979 are the area-weighted and volume-weighted mean column lengths, 〈*L*〉_area_ and 〈*L*〉_vol_, respectively, as determined from the Fourier transform of each reflection. The SRM consists of two zinc oxide (ZnO) powders: One has a crystallite size distribution centered at about 15 nm, and the other has a crystallite size distribution centered at about 60 nm. While the smaller one is well within the range that can be accessed with laboratory X-ray equipment, the 60 nm one constitutes a measurement challenge for said equipment. See Table 1 for a list of acronyms and mathematical symbols used in this paper.

This project began in 1989 and was initially patterned after the extensive body of work by Louër, Langford, and coworkers [1–7]. Data from the NIST Siemens D500 ^1^1Certain commercial equipment, instruments, or materials are identified in this paper in order to specify the experimental procedure adequately. Such identification does not imply recommendation or endorsement by the U.S. government, nor does it imply that the materials or equipment identified are necessarily the best available for the purpose. diffractometer, equipped with a Ge 111 Johansson incident beam monochromator (IBM), could be fitted using the Voigt profile shape function (PSF). Therefore the “pattern decomposition” method and analysis of crystallite size broadening outlined by Louër and Langford could be applied to our data. The manufacture of the SRM feedstock could also be patterned after the decomposition of the various precursors for cerium and zinc oxide discussed by Louër and Langford. The intent was to produce two powders; the first would consist of an isotropically broadened ≈15 nm CeO_2_ material, while the second would be a coarser, anisotropic ZnO material requiring the use of a shape model in an analysis.

The first component of the work involved the commissioning of the Siemens D500 equipped with the IBM to provide data of sufficient quality for microstructure analysis via LPA. This work was pursued over the course of 5 years, largely in collaboration with Robert Cheary [8, 9]. There were multiple difficulties with the machine; it was essentially reverse engineered in order to ensure proper performance. There were deficiencies in the supplied documentation concerning the installation and alignment of the IBM. These were addressed through personal communications with Ian Langford and the development of in-house alignment procedures for optimization of the Johansson optic. When completed, performance metrics from the machine were compared with simulations from Xfit [10], which verified that its behavior was in full compliance with the geometric models of the Fundamental Parameters Approach (FPA) to X-ray profile fitting [11]. While the results of this effort were not immediately published beyond a lengthy set of workshop notes, they were featured in the paper by Cheary *et al*. [12] on the FPA method and formed the kernel of the approach delineated in chapter 3.1 of Volume H of the International Tables for Crystallography, by Cline *et al*. [13, 14].

With the availability of quality data, additional data analysis methods were considered. This aspect of the work was pursued in collaboration with Walter Kalceff and later Nicholas Armstrong. A least-squares convolution approach was considered [15, 16] . This was followed by the development of maximum entropy and later Bayesian methods for analysis of crystallite size [17-22]. A more straightforward approach was developed using TOPAS V4.2 [23] with a least-squares convolution approach in conjunction with Mathematica [24] to generate Williamson-Hall (WH) [25] plots for qualitative evaluation of test samples in the development of the SRM feedstock.

The preparation of the SRM feedstock required the co-precipitation reactions used to prepare precursor materials to be scaled up to a considerable extent. J. J. Ritter investigated the use of a static mixer [26] for applicability as a fixed-element flow reactor to be used in continuous, co-precipitation reactions. Reactants could be pumped into the flow reactor, wherein all volume elements would undergo an identical mixing history; the primary limiting factor on batch size was the ability to wash and de-water the product. An intrepid vendor with experience in inorganic chemical preparation, GFS Chemicals, Powell, OH, was located to prepare precursor compounds of Ce and Zn. The work proceeded on a small scale using ≈50 g samples from the flow reactor, while ≈3 g specimens were decomposed in a controlled-atmosphere/vacuum tube furnace. While a suitable ZnO material was prepared with the use of the flow reactor, a management change at GFS Chemicals led to a loss of interest on their part in the project. We also investigated the decomposition of zinc oxalate [5] and were able to duplicate the results of Langford *et al*. [3]. A vacuum oven was rebuilt into a low-temperature vacuum furnace that allowed for batches of up to 125 g to be decomposed in a uniform manner.

The project proceeded with the premise that the SRM feedstock would be prepared from the decomposition of commercially available zinc oxalate. Test specimens were decomposed in the large-scale vacuum furnace at a range of annealing temperatures for the determination of the desired final annealing schedule(s). In order to gain a more quantitative understanding of the microstructure, the programs TOPAS and PM2K v1.65 [27] were also used to analyze these data [28, 29]. These whole-pattern analyses included the Warren model for stacking faults [30] to account for the *hkl* dependence of profile breadth. With the consideration of the results from these analyses, two schedules were chosen to provide two powders: One had a size distribution centered at about 15 nm, and the other had a size distribution centered at about 60 nm; these are referred to hereafter as the "15 nm" and "60 nm" materials.

Certification data were collected on the high-resolution powder diffractometer at the 11-BM beamline at the Advanced Photon Source (APS) [31] and on a NIST-built Divergent Beam Diffractometer (DBD) setup with an IBM [13]. The DBD was configured with a modern Johansson Ge 111 optic and, initially, a scintillation detector; later, it was equipped with a Bruker LynxEye XE position sensitive detector (PSD). The instrument profile functions (IPFs) of both machines were determined by means of an FPA analysis of data from SRM 660b [32] using TOPAS V5 [33]. The NIST Fundamental Parameters Approach Python Code (FPAPC) [34] was then used to simulate the IPF profiles and compute their Fourier transforms at the ZnO peak positions, using the previously determined FPA parameters. The contribution due to the crystallite size of SRM 660b was omitted from these simulations. With respect to the DBD, these simulations included a correction for the effects of sample attenuation which will broaden the observed profiles from a divergent beam diffractometer in reflection geometry. The Fourier transforms of the line profiles of SRM 1979 were extracted from the raw data sets [35]. The deconvolutions of the IPFs from the ZnO data were carried out by division. The area-weighted and volume-weighted mean column lengths (MCLs), 〈*L*〉_area_ and 〈*L*〉_vol_, were then determined from the Fourier transform of each reflection. Certified values were from the 11-BM data only. Statistical analyses of these data provided the Type A measurement errors on the certified values [36, 37].

The data from both machines were then analyzed via the FPA using TOPAS; these analyses included the Scardi and Leoni model for the crystallite size broadening [38]. The results are included as Information Values in the Certificate of Analysis (CoA). Using these values, FPAPC was used to compute the Fourier transforms of simulated FPA ZnO profiles; again, the IPF contribution was removed by division. The 〈*L*〉_area_ and 〈*L*〉_vol_ values were then computed from the transforms of each reflection. These FPA-based 〈*L*〉_area_ and 〈*L*〉_vol_ values from the 11-BM data were then compared to certified values for an assessment of the Type B, systematic measurement error. The certified lattice parameters were obtained using the FPA method via TOPAS for analyses of data from the DBD.

### 1.1 The FPA and Convolutions

The diffraction experiment is an inverse problem; the desired outcome is an understanding of a three-dimensional structure, although the observation consists only of a one dimensional data set. There are multiple structures, or solutions, that would yield diffraction data corresponding to the observation; the object, of course, is to determine the correct one. The observation of a diffraction experiment is itself a convolution of several contributions:

*I*_obs_(*x*) = *G* ⊗ *H* = *∫G*(*x* - *x՛* ) *H*(*x՛* ) *dx՛*, (1)

where *G* is the underlying shape of a reflection from the sample, *H* is the IPF at the angle at which the reflection is measured, and *I*_obs_ is the observed diffraction peak. In Fourier space, such convolutions become products. The classic statement of the convolution theorem, extended for multiple convolutions, is:

*ℱ* (*G* ⊗ *H* ⊗ *L* ⊗ *...*) = *ℱ* (*G*)*ℱ* (*H*)*ℱ* (*L*)*ℱ* (*...*), (2)

where we use *ℱ* (*f*) to represent the Fourier transform of the function *f* . It is desired to determine *ℱ* (*G*), the sample broadening function that is specific to characteristics of the specimen. From the convolution theorem we obtain the classical Stokes method [39], wherein the sample function *ℱ* (*G*) is determined by simply dividing *ℱ* (*G* ⊗ *H*), the transform of the observed lineshape, by *ℱ* (*H*), the transform of the IPF. While the technique is mathematically rigorous and appears straightforward to execute, there are, in fact, multiple difficulties. This is due to loss of statistical information with the use of the fast Fourier transform algorithm; difficulties also stem from errors due to noise, truncation, background determination, etc., that have been well documented [17, 40, 41]. Therefore, Fourier-based deconvolution methods have been largely superseded by least-squares convolution approaches in the area of microstructure analysis.

In the context of the FPA, the IPF (*H*) is split into two components: a group of geometric models representing the instrument, and the emission spectrum. The essence of the FPA is the explicit modeling of the various contributions to the geometric profile that cause the diffraction profiles to vary in shape and position as a function of 2*θ* . The emission spectrum is typically described with analytical PSFs of Lorentzian or Gaussian character. In the context of certified lattice parameter measurements, the emission spectrum provides the traceability to the International System of Units (SI) [42]. The IPF is determined with the use of a suitable standard, although the data analysis approach is dependent upon instrument geometry. The standard itself has to be crystallographically "perfect" and have a crystallite size sufficiently large as to be essentially undetectable with laboratory equipment, though not so large as to exacerbate the effects of "particle" counting statistics. With laboratory equipment configured in Bragg-Brentano geometry, models used for determination of the IPF must include sample-specific properties such as specimen transparency and specimen surface height. With high-resolution, synchrotron-based powder diffraction, the crystallite size contribution from the standard can be readily observed and removed from subsequent simulations of the IPF using the FPA. With this approach to characterization of the IPF using the FPA, the Fourier transform of the simulated IPF is set directly to *ℱ* (*H*).

### 1.2 Fundamental Description of Crystallite Size Induced Broadening

Bertaut, in his seminal paper of 1949 [43] proceeded from the simple description of a crystal as a regular lattice of atoms, to the recognition that the Fourier transform of an intrinsic line shape provides direct information about the length distribution of coherently reflecting columns of cells. We consider this development in a reduced number of steps. This section follows the notation of Bertaut fairly closely, and explanations of many of the symbols displayed are left to that work, which the reader is assumed to have at hand.

For example, eq. (4-11) of Cullity [44] describes the structure factor as a sum over the *N* atomic sites within a complete crystal:

$F_{hkl}=\sum_{n=1}^{N}f_{n}\text{exp}\left[2\pi i\left(hu_{n}+kv_{n}+lw_{n}\right)\right]\text{ Cullity (4-11).}$(3)

One sees that the amplitude of a reflected electromagnetic field can be represented as a sum over the reflection amplitudes from each of the atoms in the crystal, as long as the kinematical approximation applies. This equation can be rewritten as an integral by splitting the problem into a local integral of atomic form factors within a cell and an integral of cells over the volume of the crystals:

$G(\vec{y})=\int_{-\infty }^{\infty }S(\vec{\xi })\text{exp}\left[2\pi i\vec{\xi }\cdot \vec{y}\right]\vec{d\xi }\times \int_{v}f(\vec{x})\text{exp}\left[2\pi \vec{ix}\cdot \vec{y}\right]\vec{d}x$Bertaut (6) . (4)

The second term is *F_hkl_* for a single cell, in the form of an integral over the electron density in a unit cell, instead of a sum over individual atoms; *i.e., f_n_* for the *n*th atom replaced with *f* ($\vec{x}$), the electron density. *G*($\vec{y}$) is the scattering amplitude resulting from integrating the form factor *F_hkl_* from a single cell over the volume of the crystal, with a phase factor $\text{exp}\left[2\mathrm{\pi i}\vec{\xi }\cdot \vec{y}\right]\vec{d\xi }$ and a 'density' *s*($\vec{\xi }$) which is either 0 or 1 depending on whether the volume of interest is outside or inside the crystal. While the conversion from the sum to an integral for the left-hand term is not quite technically rigorous, it is fully plausible for materials of high crystallinity. The form of these integrals are Fourier transforms of the respective functions.

The field amplitude can then be squared (multiplied by its complex conjugate) to compute an intensity. With normalizing, expanding around the center of a cell, and shifting to a coordinate system in which *y*_1_ and *y*_2_ are perpendicular to the diffraction vector, and *y*_3_ is parallel to the diffraction vector, we find

$I=\frac{|F{|}_{0}^{2}}{4\pi |h{|}^{2}}\frac{d}{v}\int s(\vec{\xi })s(\vec{\xi }ʼ)\text{exp}\left[2\pi i\vec{\left(\xi -\xi ʼ\right)}\cdot \vec{y}\right]\vec{d\xi }\vec{d\xi }՚dy_{1}dy_{2}\text{ Bertaut (9).}$(5)

There is an intricate transformation in going to the next equation, Bertaut eq. (9') (see footnote at base of page 15 in Bertaut), that recognizes that the integrals over *ξ*_1_, *ξ*_2_, *y*_1_, and *y*_2_ share phase factors, and the transformation ends up mapping into the self-inverting property of the Fourier transform, so 

$I=\frac{|F{|}_{0}^{2}}{4\pi |h{|}^{2}}\frac{d}{v}\int s(\xi_{1},\xi_{2},\xi_{3})s(\xi_{1},\xi_{2},\xi_{3}^{ʼ})\text{exp}\left[2\pi i(\xi_{3}-\xi_{3}^{ʼ})y_{3}\right]\vec{d\xi }\text{d}\xi_{3}^{ʼ}\text{ Bertaut (9}ʼ\text{).}$(6)

The sub-integral which appears in Eq. (6),

*∫s*(*ξ*_1_*,ξ*_2_*,ξ*_3_)*s*(*ξ*_1_*,ξ*_2_*,$\xi_{3}^{ʼ}$*) *dξ*_1_*dξ*_2_, (7)

is, in fact, the area of a slice of the common volume function (since the integral over *ξ*_1_*ξ*_2_ is over the area perpendicular to the diffraction plane, and *s* is either one or zero). In Eq. (6), $\xi_{3}-\xi_{3}^{ʼ}$ is the offset between the two volumes, defined by Bertaut as *m*_3_; Eq. (7) can be rewritten in terms of this difference to yield *h*(*m*_3_), the common volume function. Its second derivative is the column length distribution.

Substituting into the angular space of diffraction, with *X* = (*θ*-*θ*_0_) *d* cos *θ*_0_*/λ* where *θ*_0_ is the diffraction angle at the center of a peak, and *d* is the lattice spacing associated with the specific *hkl*, and transforming the *ξ* variables into *m* space, we find:

*$I(X)=c\int h(m_{3})exp\left[2\pi im_{3}X\right]dm_{3}\text{ Bertaut (15).}$*(8)

$I(\theta )=c\int h(m_{3})\exp\left[2\pi im_{3}(\theta -\theta_{0})d\text{cos}\theta_{0}/\lambda \right]dm_{3}\text{ Bertaut (15) expanded. (9)}$

The common form of the Fourier transform integral, with a length scaling factor *a*, for real-space function *F*(*x*) and its transform *ℱ* (*F*), is 

$F\left(ax\right)\propto \int ℱ\left(F\right)\left(\omega \right)\exp\left(2\pi ia\omega x\right)d\omega .$ (10) 

By comparison of Eq. (10) to Eq. (9), one can see that *h*(*m*) is the Fourier transform of *I* ((*θ*-*θ*_0_) cos *θ*_0_*/λ*). Since the frequency variable in Fourier space is proportional to the reciprocal of the length scale in real space, one can see that *m*∝*λ/*cos *θ*_0_, so *m* has units of length. Then, from Bertaut eq. (19), one defines the normalized Fourier transform *t*(*m*) from *h*(*m*) as:

t(m)=-h(m)∂h∂mm→0⁺. (11)

The first and second moments of the column-length distribution, as seen parallel to the diffraction vector, are

$\bar{M}=t\left(0\right)\text{=}{❬L❭}_{area}$, (12)

$\bar{M^{2}}=\int_{-\infty }^{\infty }t(m)dm,$(13)

and thus

$\frac{\bar{M^{2}}}{\bar{M}}=\frac{1}{t(0)}\int_{-\infty }^{\infty }t(m)dm=\frac{1}{h(0)}\int_{-\infty }^{\infty }h(m)dm=❬L❭$_vol_. (14)

A point of interest, the integral of the Fourier transform of a function *f*, such as in Eq. (14) is just *f* (0), and the amplitude of the Fourier transform at *ω* = 0 is just the area of the function, so Eq. (14) is equivalent to 

$\frac{\left(\text{height of peak}\right)}{\left(\text{area of peak in }\;m\;\text{ space}\right)}\equiv \frac{1}{\text{integral breadth}}=\langle L\rangle$_vol_, (15)

which is the commonly used expression for this, and it requires no actual computation in Fourier space. It does, however, require good background subtraction from the peak to determine the height and area. Transformed from *m* or *s* (depending on the paper we are following) space, Eq. (15) becomes

$\frac{\lambda }{\cos\theta }\frac{\text{(height of peak)}}{\text{(area of peak in 2}\theta \;\text{ space)}}\equiv \frac{1}{\text{integral breadth}}={❬L❭}_{\text{vol}}.$ ^. (16)^

**Fig. 1:**
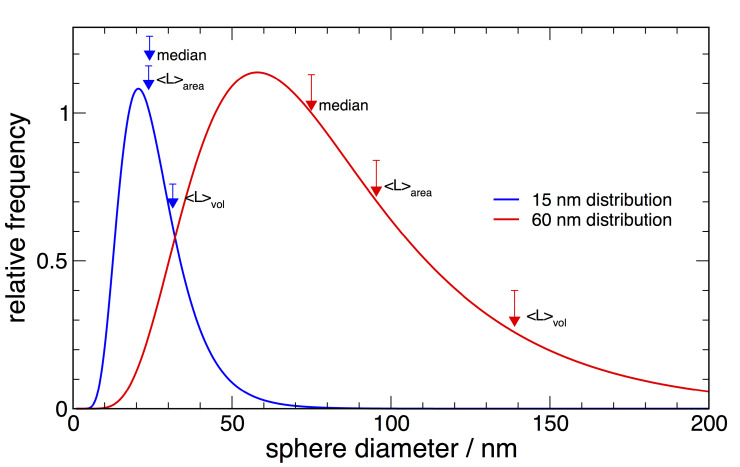
Illustration of how the various weighted measurements of mean column lengths fall on a log-normal crystallite size distribution.

Thus, the relationship between the MCL values of 〈*L*〉_area_ and 〈*L*〉_vol_ and the Fourier transform of *ℱ* (*G*) has been derived by Bertaut in a first principles context. Since the publication of Ref. [43], there have been copious publications utilizing these parameters as a means to characterize crystallite size as measured via LPA. Therefore, the certification of these two parameters for each line profile observed from the SRM 1979 materials will provide the community with well-established and accepted measurement criteria. The relationship between the 〈*L*〉_area_ and 〈*L*〉_vol_ values and the presumed log-normal crystallite size distribution is shown graphically in Fig. 1 for both the 15 nm and 60 nm materials. The methods by which these distributions and the illustrated values were obtained will be discussed in Sec. 7.

There are measurement issues associated with the determination of both the 〈*L*〉_area_ and 〈*L*〉_vol_ values. The issue with 〈*L*〉_area_ is that, from Eq. (12), is it dependent on the derivative of the Fourier transform at the origin. The computed value of this derivative is very sensitive to the values of the transform at the origin, which is also the area of the peak. A small error in the area can throw the derivative wildly off. Thus, 〈*L*〉_area_ is intrinsically harder to calculate correctly than 〈*L*〉_vol_. However, 〈*L*〉_area_ is relatively insensitive to the instrumental aberrations, since it only samples data at low Fourier frequency, where the Fourier transform of the IPF is near unity. On the other hand, 〈*L*〉_vol_ is an integral over all frequencies, including data at high frequency, so it is sensitive to errors in the IPF and to noise which is amplified by the IPF deconvolution.

## 2 Data Collection and Preliminary Analysis Methods for Feedstock Selection

Data for the evaluation of feedstock candidates were collected on a Siemens D500 diffractometer equipped with a Johansson Ge 111 IBM, sample spinner and scintillation detector. Copper *Kα*_1_ radiation was used. The D500 was configured with a divergence slit of 0.67º; the receiving optics included a slit of 0.05º and 2º Soller slits. Data from SRM 660a, LaB_6_ [45] were collected to determine the IPF; the collection and analysis were done in a manner analogous to that described in Ref. [13]. Data from the ZnO and CeO_2_ test specimens were collected in continuous scans from 25º to 125º 2*θ* with a step width of 0.02º and a count time of 16 s to yield a total scan time of approximately 24 h.

Data analysis methods used in the preliminary studies consisted primarily of WH plots. Data from SRM 660a were fit with the Split Pearson VII PSF. These fitted parameters, full width at half maximum (FWHM) and coefficients, were modeled as a function of 2*θ* using codes written in Mathematica. TOPAS was then used to perform a least-squares convolution approach using a Pearson VII PSF as the specimen broadening function. Mathematica codes were then used to process the breadths of these profiles to generate the WH plots.

Once the number of candidate materials suitable for the SRM feedstock had been narrowed, a more complete understanding of the microstructure was realized with the use of two whole powder pattern methods. The first was the FPA-based Rietveld [46, 47] analyses using TOPAS, and the second was whole powder pattern modeling (WPPM) [48] via PM2K. However, the data from the D500 were less than ideal for analyses with either the FPA or WPPM methods because they both require the modeling of the emission spectrum. As discussed in Ref. [13], the early Johansson optic of the D500 produced an asymmetric emission spectrum that defied accurate modeling; see Fig. 29 in Ref. [12] vs. Fig. 13 in Ref. [13]. However, given the preliminary and qualitative nature in which these results were to be used, this shortcoming was considered acceptable.

The energies of the Cu *Kα*_1_ emission spectrum as characterized by Hölzer *et al*. [49] were used with both analysis methods. Parameters specific to the IPF were modeled using data from SRM 660a and fixed in subsequent refinements. With the FPA, the emission spectrum from the Johansson optic was modeled with a series of Gaussian profiles, three for the *Kα*_11_ line, and a fourth for the *Kα*_12_ line. The breadths and intensities of these profiles were allowed to refine. The "full" axial divergence model [50, 51] was used with the two Soller slit values being refined as a single value. Other refined parameters included the incident slit angle, scale factor, Chebyshev polynomial terms for modeling of the background, the lattice parameters, specimen displacement and attenuation terms, and a term for Lorentzian size broadening. With the analyses of ZnO, the Warren model for stacking faults in a hexagonal close packed (HCP) structure was included to account for the *hkl* dependence on profile breadth. The model includes two refinable parameters, *α*, which is proportional to the density of the deformation faults, and *β*, which is proportional to the density of the growth faults. A Lorenzian strain term in tan*θ* was also included.

With the use of PM2K, the IPF was modeled using four pseudo-Voigt PSFs, three for the *Kα*_11_ line, and the fourth for the *Kα*_12_ line. The breadth, intensity, and relative contributions of the Lorentzian and Gaussian components were refined. The *U*, *V*, and *W* parameters of the Caglioti function [52] were refined to model the FWHM dependence on 2*θ* and the Finger [53] model was used to account for profile asymmetry. Lattice parameters were fixed at the certified values for SRM 660a; the effects of specimen displacement were modeled. With the WPPM analyses of the ZnO, the specimen broadening function was specific to modeling of cylinders with a crystallite size distribution presumed to be log-normal [38]. Other refined parameters were analogous to those used with TOPAS.

## 3 Preparation of Feedstock

In order to prepare bulk quantities of precipitated materials, the services of a chemical manufacturer were secured to set up and operate a fixed-element flow reactor. The application of a static mixer for use as a flow reactor was pioneered by one of the authors, J. J. Ritter. The flow reactor itself consists of a tube that contains a series of stacked, stationary geometric elements designed so as to interact with the reacting fluid stream flowing through the tube. The elements cause the stream to divide, swirl, and recombine in a mathematically predictable fashion. The overall effect is to give all of the processed material nearly the same velocity, residence time, and degree of radial mixing. As a result, each increment of product exiting from the reaction tube has had an identical and uniform mixing history. The potential for preparing large amounts of uniform powder using this technique is limited only by the amounts of reactant solutions available, the durability of the pumps used to drive the solutions through the reactor, and the inclination of the operator to wash the resulting precipitate so as to be free of the reactant residue.

Initially, small samples of up to 50 g of the precursor compounds were prepared using the flow reactor. These precursor materials were decomposed in a small-scale controlled atmosphere/vacuum tube furnace using a heating schedule that was, at least initially, based on the work of Louër *et al.* Small, 3 g to 5 g quantities of the oxide were produced in this manner. The routes investigated with this approach consisted of cerium(IV) sulfate, Ce(SO_4_)_2_, and cerium(IV) ammonium nitrate, Ce(NH_4_)_2_(NO_3_)_6_, both precipitated with aqueous ammonia, NH_4_OH. The routes investigated to prepare ZnO included zinc nitrate, Zn(NO_3_)_2_, and zinc acetate, Zn(OOCCH_3_)_2_, again, both precipitated with aqueous ammonia, NH_4_OH. While initial thermal decomposition treatments were performed in air or under N_2_, it was found that decomposing the material under a vacuum often yielded more desirable results.

It was found that both the cerium and zinc nitrate preparations yielded unacceptable results due to either uncontrollably rapid growth of the crystallites and/or the development of crystalline defects leading to microstrain broadening. The ex-sulfate preparations of CeO_2_ yielded material that exhibited promising diffraction data. However, the precipitate apparently gelled upon exiting the flow reactor, and upon decomposition, it yielded large polycrystalline shards of several hundred micrometers in size. An extensive effort was undertaken to address the difficulty, which included modifications to the chemistry as well as the use of a high shear rate mixer; success was not realized. Through a correspondence between the author and D. Louër (personal communication), it was determined that the CeO_2_ used for the Commission on Powder Diffraction round robin [54] was successfully prepared in a nonaqueous manner. Cerium sulfate was simply added to the ammonia solution; this method could not be scaled up to the kilogram level, and the effort concerning the preparation of CeO_2_ was abandoned. The thermal decomposition of the flow reactor-prepared zinc acetate yielded a desirable material. With extensive experimentation, the fairly complex time/temperature profile required to decompose the ex-acetate ZnO into a phase pAure powder of the desired crystallite size range was developed. The last route that was considered was the preparation of ZnO powder from the decomposition of a commercially available oxalate precursor, as per the method and results of Auffrédic *et al*. and Langford *et al*. [3, 5]. Our experiments yielded an acceptable material that was in correspondence with the work of Langford *et al*.

Following the preliminary work on the experimental quantities in the tube furnace, the large-scale vacuum furnace, capable of decomposing 125 g lots of material at a time, was commissioned. The primary virtue of this device was that it could uniformly heat a large powder bed of material, under vacuum, with good temperature control, through the 50 ºC to 500 ºC region. The furnace itself started out life as a vacuum oven with an internal volume of approximately 0.03 m^3^. Two conventional resistance heating elements, each approximately 0.3 m × 0.3 m in dimension, were installed in a parallel configuration approximately 25 mm apart. In this gap would reside the boats that contained the precursor to be decomposed. Temperature control was accomplished with a conventional proportional-integral- differential (PID) controller. Two Type K thermocouples were installed, one for the temperature controller and a second to permit continuous monitoring of the temperature profile. Vacuum was provided with a roughing pump; 25 mm diameter vacuum lines, including a cold trap, provided for improved conductance to reach a vacuum of 20 Pa (0.15 torr). Temperature control was excellent in the region of 75 ºC to 500 ºC, with variations outside the desired control temperature typically being less than 2 ºC [29].

**Fig. 2:**
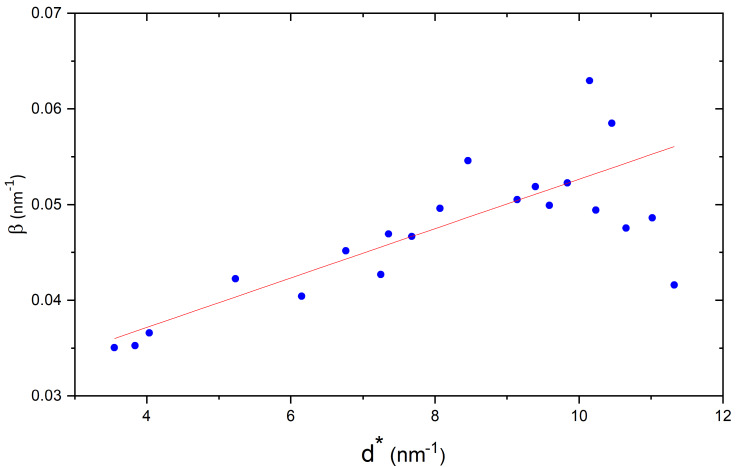
Williamson-Hall plot of acceptable ex-acetate ZnO prepared with the flow reactor.

**Fig. 3:**
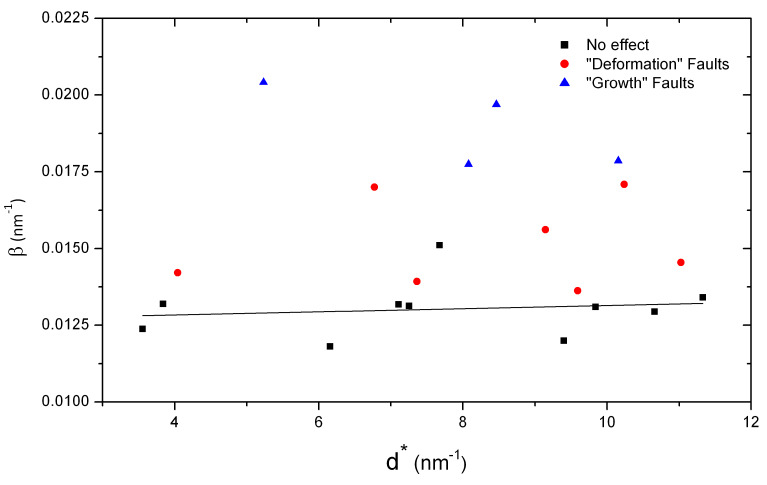
Williamson-Hall plot of ex-oxalate ZnO exhibiting the effects of stacking faults as reported by Langford *et al [**3**]*.

Again, a series of experiments was performed using a small volume of material to duplicate the earlier work using this larger furnace, and to establish the specific relationship between particle size and processing parameters. A WH plot is shown in Fig. 2 for the ex-acetate ZnO, decomposed under vacuum up to 150 ºC, followed by a second calcination in air to 400 ºC. The data in Fig. 3 are consistent with a desirable nanocrystalline ZnO of a suitable crystallite size and limited microstrain broadening. Analysis via Transmission Electron Microscopy (TEM) confirmed that the material was well dispersed, consisting largely of single-crystal particles. Unfortunately, while the material did show great promise for use as the SRM artifact, the chemical firm that worked with us on the project lost interest. We could not realize the preparation of ex-acetate ZnO for the SRM in a timely manner.

It was decided to proceed with the ex-oxalate ZnO as the feedstock for SRM 1979. The precursor zinc oxalate powder, 99.999% pure (metals basis), was obtained from Alfa Aesar (Ward Hill, MA). Initial experiments duplicated the time/temperature profile of Auffrëdic *et al*.; the material was heated in the vacuum furnace, rapidly from room temperature to 70 ºC, and then from 70 ºC to 0 110 ºC at a rate of 2 ºC */*h followed by another rapid increase to 250 ºC and then up to 335 ºC at 2 ºC */*h followed by cooling to room temperature. In order to assess the effects of annealing temperature on microstructure, small quantities of ZnO from the vacuum experiment were then heated in air to temperatures ranging from 400 ºC to 500 ºC at 25 ºC intervals, with a final lot being annealed to 550 ºC. The heating rate for these experiments was 2 ºC */*h, and the specimens were quenched by a withdrawal from the furnace when the final temperature was reached.

A WH plot from the ex-oxalate ZnO processed to 400 ºC is shown in Fig. 3. These results are in correspondence to those reported by Langford *et al*, and the impact of the stacking faults is illustrated. The observed broadening can be classified according to *hkl*, indicative of the nature of broadening that occurs in HCP materials due to stacking faults. The peaks can be segregated into three groups. Group 1 contains *hk0* peaks, and peaks with *h*-*k* = 3*n*, exhibiting no broadening effect. Group 2 has peaks with *h*-*k* = 3*n*±1, *l* odd, that exhibit deformation or strain broadening. Finally group 3 has peaks with *h*-*k* = 3*n*±1, *l* even, that exhibit broadening due to growth faults [5, 30]. The slope of a linear fit to the peaks in group 1 is about 5×10^-5^, indicating negligible strain, and the intercept of the fit is 0.0126, giving an 〈*L*〉_vol_ of about 80 nm.

**Fig. 4:**
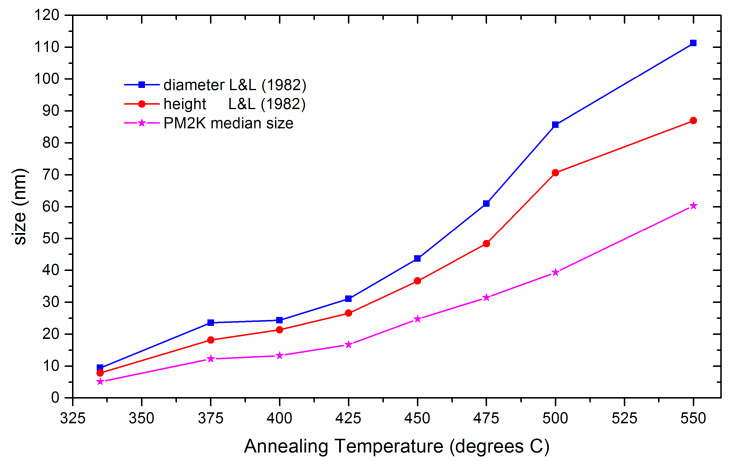
Crystallite size, expressed as the cylinder diameter and height (from Langford and Louër [55]), and median crystallite diameter (from PM2K) of the ex-oxalate ZnO as a function of annealing temperature.

**Fig. 5:**
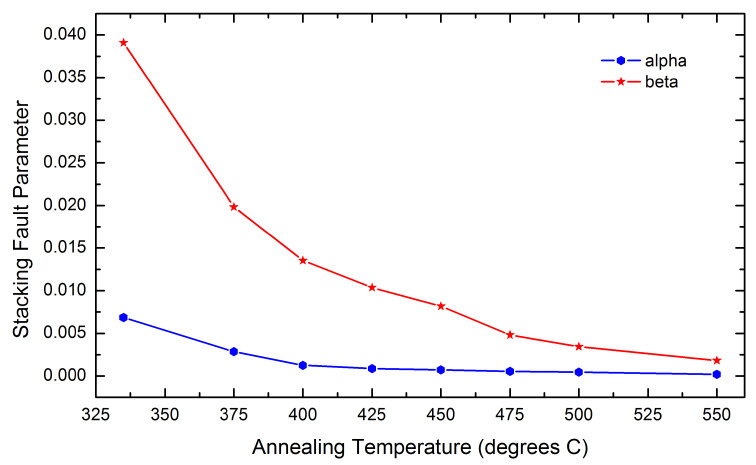
Variation of stacking fault parameters *α*, proportional to the density of deformation faults, and *β*, proportional to growth faults, of the ex-oxalate ZnO as a function of annealing temperature.

While the WH method was of sufficient rigor to establish the validity of the processing approach, we embellished it with the use of a cylindrical shape model [55]. The method assumed that all peak broadening was due to particle size effects, i.e., no strain broadening, and that the hexagonal ZnO crystallites were well represented by a cylinder. Equations 10 and 17 from Ref. [55] were then fit to extract the cylinder diameter (*D*) and height (*H*). The result of this analysis as applied to our investigation of the effect of processing temperature on crystallite size is shown in Fig. 4, where we see that the crystallite size ranges from 10 nm to 110 nm. The disc-like morphology of the crystallites is verified by these data, as the average aspect ratio, *D/H*, is 1.1 for the smaller crystallites and 1.25 for the larger crystallites. This is consistent with the results from Auffrédic *et al.* [5]. TOPAS was used to determine the dependence of stacking fault density on annealing temperature, as displayed in Fig. 5. The relatively small values for *α* reflecting the low density of "deformation" faults throughout the temperature range and the fact that there are no faults of this type above the annealing temperature of 400ºC. The values for *β* indicate a substantial level of "growth" faults at the lower temperatures, but they too are absent at the annealing temperature of 550 ºC.

**Fig. 6:**
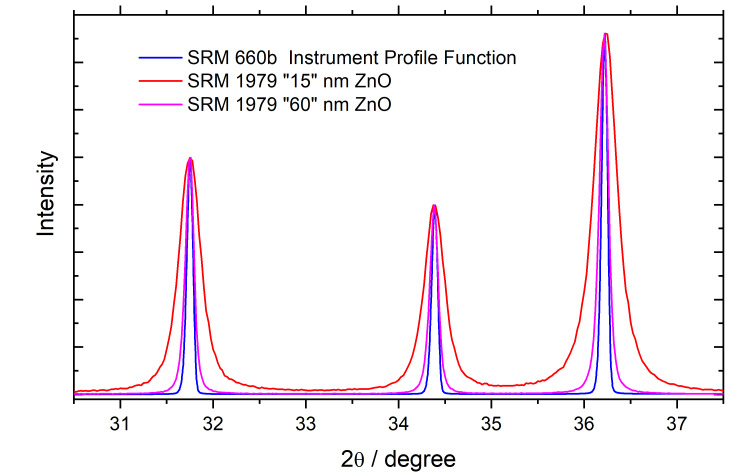
Profiles from the two ZnO powders of SRM 1979 collected on the DBD, with simulated profiles of the IPF, via SRM 660b.

These data were then analyzed with the WPPM approach as implemented in PM2K. The median crystallite sizes, using models for a log-normal size distribution and a cylindrical crystallite shape, are plotted as a functional of annealing temperature in Fig. 4. The discrepancies between these results and those from the WH analyses are not unexpected, because the parameters reflect entirely different metrics of crystallite size; the trends indicated by the two techniques are identical, however. The analyses with PM2K also indicate that the crystallites are in the form of discs with a *D/H* ratio of 1.4. It was decided to prepare the SRM feedstock using the two annealing temperatures of 400 ºC and 550 ºC. The final annealing schedule was as follows: The material was heated in the vacuum furnace, rapidly from room temperature to 70 ºC, and then from 70 ºC to 110 ºC at a rate of 2 ºC */*h, followed by another rapid increase to 250 ºC, then up to 400 ºC at 2 ºC */*h, and finally a slow, i.e., no power to the heating elements, return to room temperature. This material was then divided into two lots for each of the two size ranges. Material was then loaded into a conventional furnace, with no atmospheric control, that was rapidly heated to a temperature of 350 ºC. For the 15 nm crystallite size, it was heated at a rate of 2 ºC */*h to a final temperature of 400 ºC, while, for the 60 nm crystallite size, an identical rate was used with the final temperature being 550 ºC. Samples were immediately removed from the furnace once the final temperature was reached. An example of the diffraction data from these two materials collected on the NIST DBD, with the illustrated IPF profiles being synthesized via the FPA, is shown in Fig. 6.

## 3.1 Characterization with Dynamic Light Scattering and TEM

**Fig. 7:**
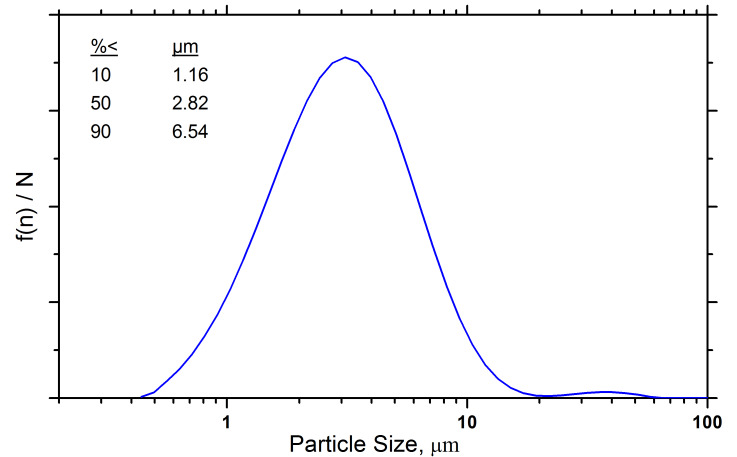

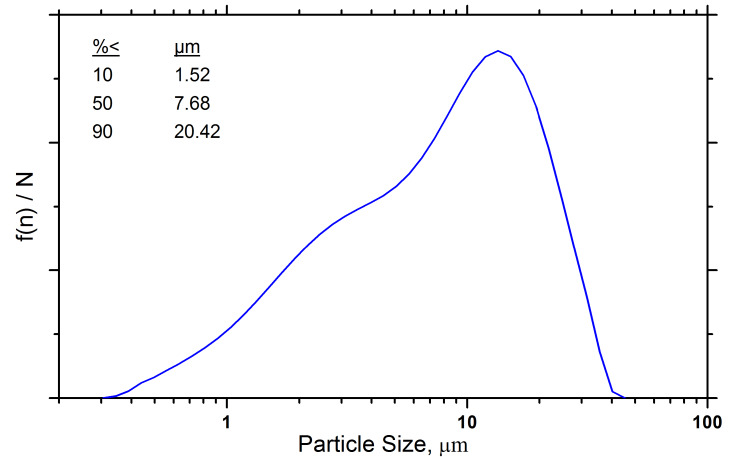
Particle size distributions measured with dynamic light scattering for (a) the 15 nm ZnO material and (b) the 60 nm ZnO material.

The particle size distributions of the two SRM materials were measured with dynamic light scattering. The results are shown in Fig. 7. These data indicate that the particle size of these two powders is acceptable for their use as powder diffraction specimens. The slight hump in the 15 nm material at 40 *µ*m is real, but it is not overly problematic. Experiments indicated that a low-intensity kneading operation with a mortar and pestle will eliminate these agglomerates without an impact on the crystallite size. The distribution of the 60 nm material is bi-modal, with a small amount of material in the 30 *µ*m to 40 *µ*m range, which is not problematic.

**Fig. 8:**
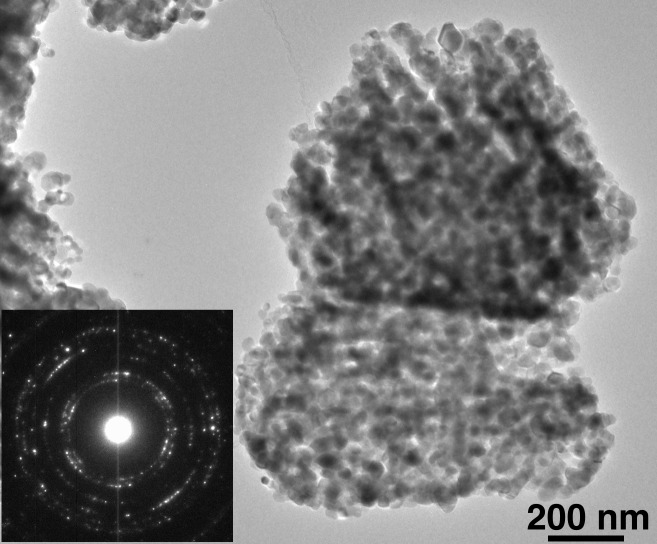

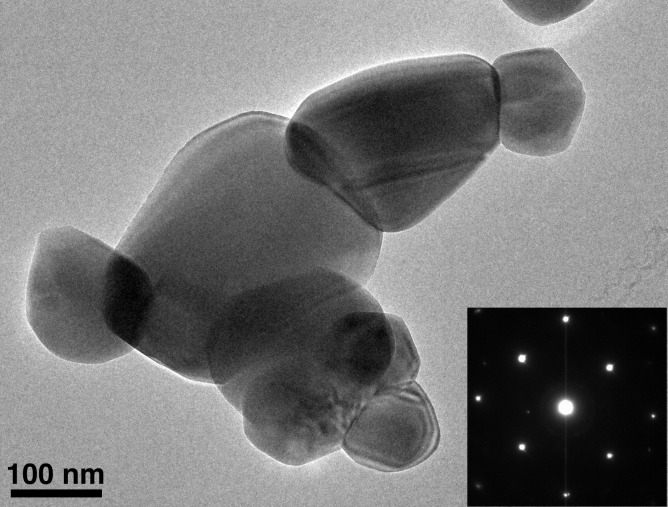
TEM images and diffraction patterns from the ZnO feedstock for (a) the 15 nm ZnO material and (b) the 60 nm ZnO material.

Images and diffraction patterns obtained from analyses using a TEM are shown in Fig. 8. The clearly polycrystalline nature of the 15 nm powder is illustrated in Fig. 8 (a); furthermore, the diffraction pattern is consistent with the crystallites displaying a fair degree of texture. This suggests that the parent zinc oxalate crystals were quite large and upon decomposition formed the observed polycrystalline aggregates. The stacking faults within the crystallites are also apparent in this image. The diffraction pattern from the 60 nm powder indicate a single crystal. Unfortunately, a quantitative assessment of crystallite size distributions for either powder would be problematic with a TEM owing to the state of aggregation of the crystallites.

## 4 Data Collection

As aforementioned, data for the certification of SRM 1979 were collected using two instruments, the NIST DBD and the diffractometer located at APS 11-BM. The high resolution of the 11-BM machine rendered it the appropriate choice for data to be used for certification of MCL values. Data from the NIST DBD were used for certification of lattice parameters, because the machine has an accurate goniometer, its optics are well understood and its emission spectrum is traceable to the SI. Data from the DBD were also used for homogeneity verification, because this requires a large number specimens to be run and measurement precision is the only critical issue for this verification. The data from the DBD were collected with the use of two detectors. One complete data set was collected using a scintillation detector, and a second set was collected using the PSD. This was due in part to the fact that the PSD was being commissioned concurrently with the pursuit of this work.

Ten units of SRM 1979 were removed from the population of 200 in accordance with a stratified random protocol. For data collection on the DBD, two specimens were prepared from each bottle of the two materials, 15 nm and 60 nm, of SRM 1979, for a total of 40 specimens. Both the order in which the specimens were prepared and the run order were randomized. Data for the certification of lattice parameters were collected using zero background quartz plates as specimen holders to eliminate the sample absorption correction. Samples were prepared by kneading a small amount of material in a mortar and pestle using ethanol as a suspending agent. Small amounts of the suspension were transferred to the quartz plate and allowed to dry, resulting in a thin film of the zinc oxide powder. With the 11-BM machine, data were collected from five randomly selected specimens of the 15 nm material, while data were collected from four specimens of the 60 nm material. Prior to data collection on the zinc oxide, a data set from SRM 660b was collected, and midway through the data collection, a second data set on the SRM 660b was collected, and a third run was performed when the data collection from the zinc oxide was complete. The run order of the SRM 1979 samples was randomized.

## 4.1 NIST DBD with the PSD

For this data collection, the 1.5 kW copper tube (8.047 keV) of fine focus geometry was operated at a power of 1.2 kW. The variable divergence incident slit was set to 0.9°, and a 0.2 mm (0.05°) receiving slit was used. The receiving optics were fitted with a 4.4° Soller slit. The diffractometer was scanned in 2*θ* steps of 0.02°, and the count time was 16 s. The scans were from 25.0° 2*θ* to 125.0° 2*θ*. This resulted in a scan requiring just under one full day to complete. Samples were spun at 0.5 Hz during data collection. The machine was located within a temperature-controlled laboratory space where the nominal short-range control of temperature was ±0.1K. The temperature was monitored using two 10 kΩ thermistors with a Hart/Fluke BlackStack system that was calibrated at the NIST temperature calibration facility to ±0.002 °C [56]. The source was allowed to equilibrate at operating conditions for at least 1 h prior to recording any data. These data were used for homogeneity verification from an analysis of lattice parameter values.

## 4.2 NIST DBD with the PSD

For these data collections, the instrument was otherwise configured and operated as per the use of the scintillation detector. Axial divergence was limited by a 1.5º Soller slit fitted to the entry window of the PSD. The PSD was scanned using multiscale stepping, with the major step size of 16 pixels and a minor step size of 1*/*4 pixel, where a pixel is 0.0198º, Each minor step was counted for 2 s, resulting in 24 s of dwell time on each pixel, and each spectral feature being measured at 12 points along the face of the detector. The total data collection time for these runs was about 3 h. The machine was equipped with an automated antiscatter slit located above the sample to prevent air scatter from the incident beam from entering the PSD and contributing to the low-angle background level. Its height above the specimen varied as *αR/*(2 cos *θ*) where *α* is the full equatorial divergence angle of the incident beam. These data were used for homogeneity verification from an analysis of 〈*L*〉_area_ and 〈*L*〉_vol_ values, determination of information values concerning microstructure, and the certification of the lattice parameters.

## 4.3 APS 11-BM

The sample was mounted in a kapton capillary of 0.8 mm diameter and spun at >90 Hz during data collection. The wavelength was set to 0.0414217 nm (approximately 30 keV); this value was verified in our analysis using data from SRM 660b. The scans were taken from 0º to 40º 2*θ* in 0.001º increments. Each step was counted for 0.1 s. One of the SRM 660b scans ranged from 0º to 110º; this was used for a microstructure analysis of the LaB_6_ of SRM 660b.

## 5 Extraction of Transforms

The primary historical difficulty in using Fourier methods for analyses of powder diffraction data has been the determination of the background. The procedure we outline is to fit the data in a two-step process. The data are first fit with an analytical PSF such as a Voigt, with weighting altered so as to favor the accurate fitting of the tail region of the peak. This ensures the accurate modeling of the background at the expense of the quality of the fit to the center portion of the profile. The residuals of the fit are then addressed in a second step wherein they are recombined with the fitted PSF itself. This method effectively solves the infamous "hook" problem in the Fourier transform analysis of diffraction patterns [57].

### 5.1 Characterization of the IPF

To obtain the IPF, *g*_IPF_ (which for brevity is here called *H*, per Eq. (1)), and its transform *ℱ* (*H*), we apply the FPA to high-quality data sets of SRM 660b measured on the two instruments. By a nonlinear least-square (NLLSQ) fit of the SRM 660b data to an FPA model, the parameters needed to compute peak shapes at any angle are obtained. Since the FPA-generated line shapes are produced by a convolution of the various aberrations, and this convolution is carried out in Fourier space in the FPAPC, the Fourier transform of the IPF, *ℱ* (*H*), can be computed directly. With this approach, *ℱ* (*H*) is noiseless, since it is derived from a theoretical model, and it is a continuous function, *i.e.*, it can be calculated for any Fourier frequency. It does, though, have systematic uncertainties attached to it as a result of uncertainties in the FPA parameters that limit the ability to carry out this division in regions where *ℱ* (*H*) is small but may have a large relative systematic uncertainty. However, the statistical errors in *ℱ* (*G*⊗*H*), determined from linear least-squares fitting of the data, can be also scaled by *ℱ* (*H*). The resulting coefficients can be used in weighted least-squares fitting to compute correct statistical errors on parameters derived there from. This approach is then more complete than the Stokes method, which does not carry forward the errors for weighted fitting. With the inclusion of an IBM on the laboratory instrument, the emission spectrum is limited to the Cu *Kα*_1_ profile alone, substantially reducing the complexity of the spectral component of IPF and aiding reconstruction of the intrinsic line profile from the equipment and analyte.

Data were analyzed using the FPA method as implemented in TOPAS with a Pawley [58] analysis. Data from the DBD were analyzed as per the procedure outlined for the D500, Sec. 2, except that SRM 660b was used for determination of the IPF. However, with the modern characterization of the Cu *K α* emission spectrum [59] and the development of the "band-pass" model for characterizing the effects of the Johansson optic [60], it was thought appropriate to re-analyze the data to be used for informational microstructural values and lattice parameter certification from the DBD.

The band-pass model has three parameters that can be refined: *δ*_3_ and *δ*_0_ set the width and shape of the band-pass, respectively, while *u*_0_ is a tuning parameter that sets the position of the window relative to the position of the *Kα*_1_ line. The first two parameters are specific to the crystal itself and, after initial determination, are essentially invariant. The third is specific to the diffraction angle of the Johansson optic which is known to drift by small amounts. Refinements of the band-pass parameters were accompanied by other IPF-specific parameters such as divergence and Soller slit angles. The IPF of the DBD, a divergent beam machine of reflection geometry, will also be affected by sample attenuation. Therefore, simulations of the IPF via FPAPC included values for attenuation of the ZnO obtained from FPA refinements of ZnO data from the DBD.

With the FPA analysis of SRM 660b to determine the IPF of the 11-BM machine, the lattice parameter's value was fixed to the certified values. The incident beam spectrum was also modeled with the use of three Gaussian profiles of a common, refined wavelength; breadths and intensities were refined independently. The incident beam was considered parallel in the equatorial plane. The "full" axial divergence model was used, again with the two Soller slit values being refined as a single value. This is not technically correct, because the 11-BM machine is not symmetric in the context of the incident vs. diffracted beam path lengths. A quality fit was obtained nonetheless; other, more complex modes of refinement were tested with no improvement. With their refinement, the lengths of the "filament," sample, and "receiving slit" were constrained to a common value. Slight variations were observed in peak position; these were modeled with a second-order polynomial for a substantial reduction in residual errors. The terms refined were essentially constant across SRM 660b data-sets, leading to the conclusion that the goniometer was the origin of this issue. The effect is essentially undetectable unless the sample exhibits minimal broadening. While crystallite size broadening was nearly undetectable for the DBD, it amounted to a substantial portion of the apparent IPF for the 11-BM machine. The scan to 2*θ* = 110º on 11-BM allowed for a robust refinement of the crystallite size contribution from SRM 660b. A value for 〈*L*〉_vol_ of 500 nm was realized. This value is consistent not only with our knowledge of this material, but also with refinements of SRM 660b data collected from the more limited 2*θ* range. The "true" IPF of a machine does not include this crystallite size contribution; therefore, it has been omitted from subsequent computations of the IPF from the FPA parameters.

**Fig. 9:**
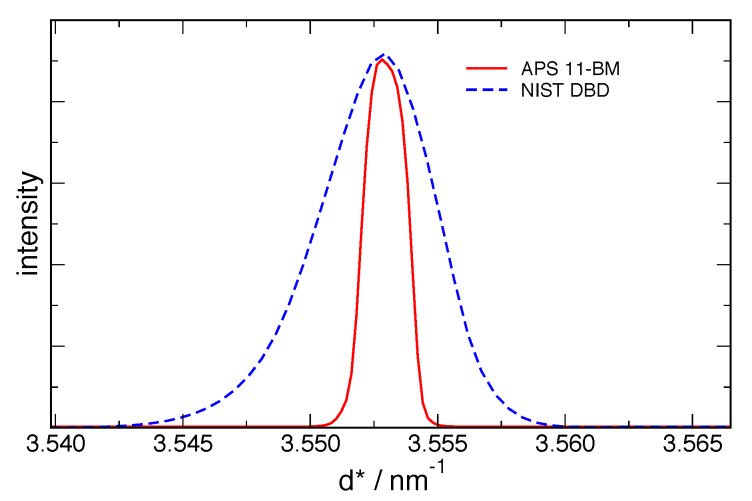
Real space representations of the IPF from the NIST DBD and APS 11-BM.

**Fig. 10:**
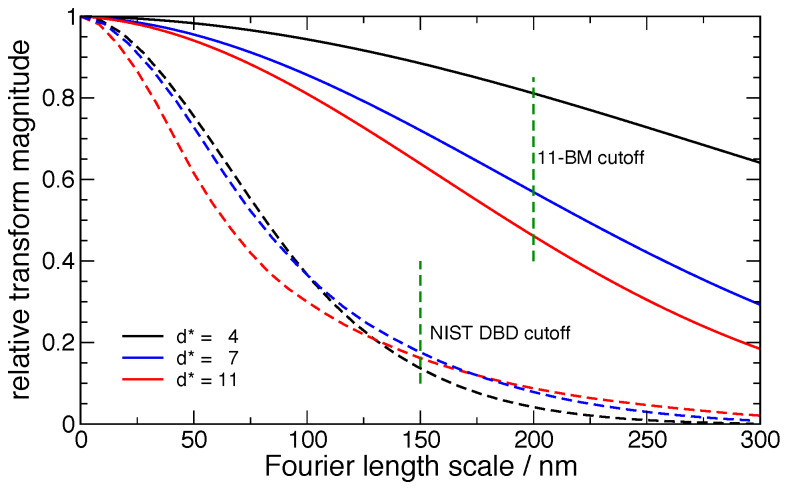
Fourier transforms of the IPF *ℱ* (*g*_IPF_) from the NIST DBD and APS 11-BM.

The functional behaviors of the IPFs for both machines, computed from FPA parameters, are shown in Fig. 9 for real space, 110 reflection, and in Fig. 10 for Fourier space. Since the transforms of the observation are divided by those of the IPF, regions in which the IPF is very close to unity are relatively insensitive to the instrument, but regions where the IPF is small have large corrections. This correction increases both the statistical noise from the data and the systematic error associated with the uncertainty in the IPF itself. The highly collimated beam, narrow energy spread, and capillary sample geometry of 11-BM, in contrast to the axial divergence and flat specimen effects of the DBD, afford the 11-BM the much higher resolution apparent in both figures. The much higher response of 11-BM at high Fourier frequencies (long length scales) is apparent in Fig. 10 relative to that of the DBD. As will be discussed subsequently, the cutoffs shown in Fig. 10 are, in the case of the DBD, due to noise in the transform of the IPF, and in the case of 11-BM, due to a lack of any additional information available from the sample above the Fourier length scale of 200 nm.

### 5.2 Profile Fitting

**Fig. 11:**
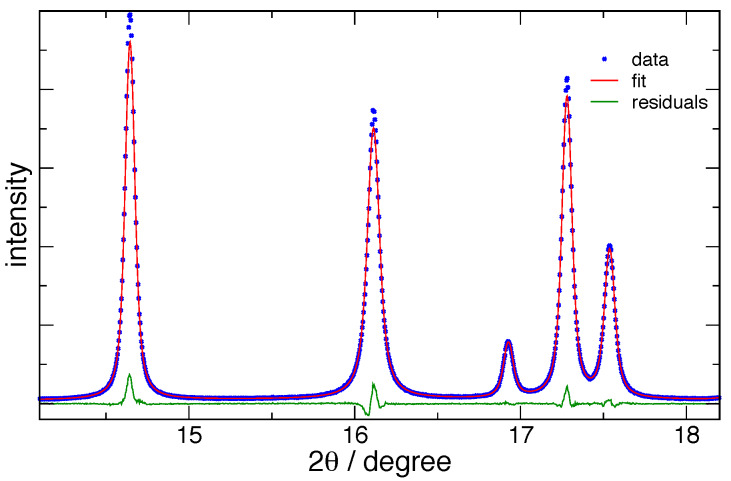

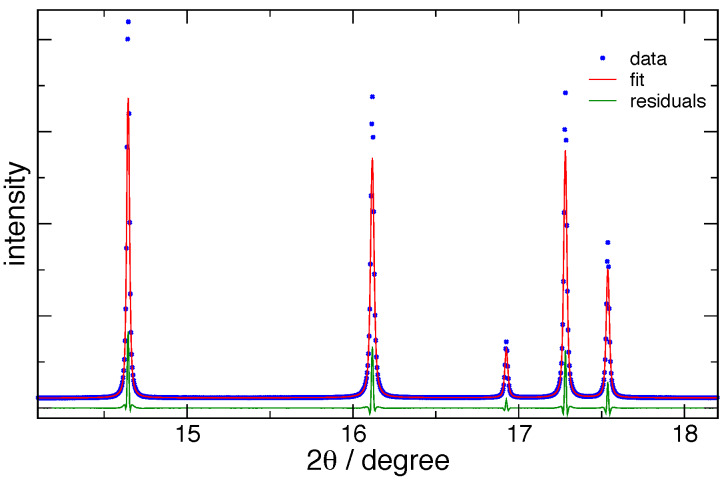
Typical region around peaks, from 11-BM, for (a) wide peaks from 15 nm ZnO material and (b) narrow peaks from 60 nm ZnO material. Blue crosses indicate data, red curves are the fit, and green curves are the residuals. Note that the residuals around the peaks have very short tails, and are easily isolated.

For the analysis of SRM 1979, Voigt PSF functions were used as they provided the best initial fit to the data. The profiles were generated numerically by the algorithm of Mendenhall [61]. For normal Poisson-statistics least-squares fitting, one weights the data with *w* = 1*/σ* ^2^ = 1*/y*, where *y* is the number of counts in a bin of the pattern. With this analysis, the data are weighted with *w* = 1*/*(*y*_0_ + *y* + *a*^2^*y*^2^), which, with *y*_0_ being a few counts (to avoid divide-by-zero if an empty bin arises), and *a* = 0.1, places more emphasis on the bins with a small number of counts, and less on the peaks. Using the Voigt functions with the weighting described above, and a background consisting of the first 8 Chebyshev polynomials in 2*θ* and a term in 1*/*2*θ*, we carried out a NLLSQ fit to the entire pattern to compute a good approximation to the shape of the pattern everywhere except on top of the peaks. The curves in red in Fig. 11 are examples of the result of this fit.

The residuals of the resulting fit, as seen in the green curves in Fig. 11, were then divided into specific regions of width Δ2*θ* around each peak where they were non-zero. We then computed, by weighted least squares, a representation of the residuals. By choosing the basis set of this fit to be trigonometric, it can be immediately interpreted as a Fourier series. This resulting Fourier series can then be summed with the Voigt transform to produce a final transform associated with a specific reflection. Thus, the shape of a peak is represented as:

*f* (2*θ*) = *C* (Δ2*θ*) Voigt(*w*,σ;2*θ*-2*θ*_0_) + *A*_0_ (17)

$+\sum_{k=1}^{n}A_{k}\cos k\omega_{0}\left(2\theta -2\theta_{0}\right)$

$+\sum_{k=1}^{n}B_{k}\sin k\omega_{0}\left(2\theta -2\theta_{0}\right)$

where the *A_k_* and *B_k_* come from the least-squares fit to the residuals, and 2*θ*_0_ is the nominal peak center. The factor (Δ2*θ*) normalizes the area so that the constant *C* in Eq. (18) is identical to that in Eq. (17).

Equation (17) separates the data into an analytic function, which extrapolates the tails of the peak to infinity, and the residuals, which, when recombined with the analytic function, fully reconstruct the data. The extrapolation of the tails to infinity enables a correct computation of the area of the peak, which then makes it possible to compute the Fourier transform without distortion due to real-space truncation [40]. The residuals carry with them statistical uncertainties from the counting statistics of the original data. The resulting Fourier transform of the peak will be the "ideal" noiseless transform, plus a uniform white noise component at all Fourier frequencies, which is the Fourier transform of the statistical noise. When the deconvolution is carried out, as described below, it amplifies the high frequencies, resulting in increasing noise at high frequencies. If no accounting is made for this noise in subsequent data analysis, results can be very unstable. However, if the Fourier transform is carried out by linear least-squares fitting, such that the statistical uncertainties can be computed for the Fourier coefficients, then these uncertainties can also be scaled by the same deconvolution kernel as the data. In this case, if subsequent analysis is carried out in a manner aware of the uncertainties, *e.g.*, by another stage of linear least-squares fitting, the fit will appropriately de-emphasize the noisy tails and will remain as stable as possible based on the less noisy regions of the data.

### 5.3 Computation of Transforms

The discrete Fourier transform of Eq. (17) is: 

*ℱ*$(f)(k\omega_{0})=C\exp\left[-w\left|k\omega_{0}\right|-\frac{k^{2}\sigma^{2}\omega_{0}^{2}}{2}\right]+A_{k}+iB_{k}.$ (18)

The raw Fourier transforms thus produced contain both the needed information about the material, and the IPF, which is to be removed. Since the material peak shape and the IPF are combined by convolution, in the Fourier domain, the transforms are multiplied, as per Eq. (2). To remove the IPF, we divide by its transform, which is discussed in Sec. 1.1.

This IPF can then be interpolated onto the same *ω* grid as used in Eq. (18), and the division carried out. Thus, if *ℱ* (*g*_IPF_)(*kω*_0_) is the transform of the IPF, (where *g*_IPF_ is more generically called *H* in Eq. (1)), then

**Fig. 12:**
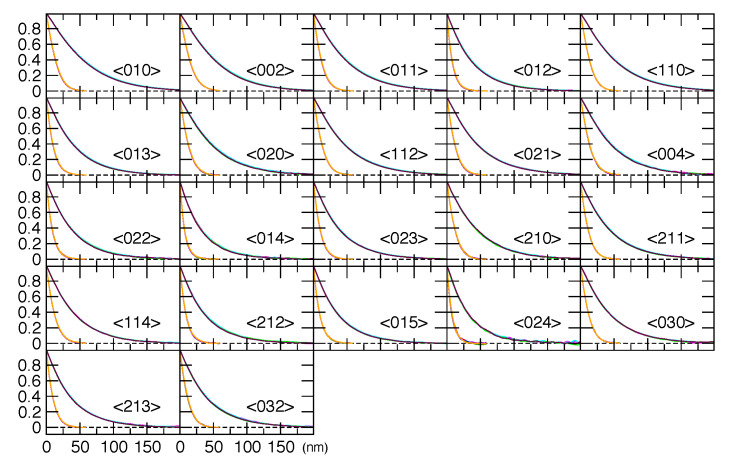
Fourier transforms of individual ZnO reflections from the 11-BM data. The two clusters of data correspond to the two crystallite sizes. The numbers in angle brackets are the *hkl* indices for the reflection.

**Fig. 13:**
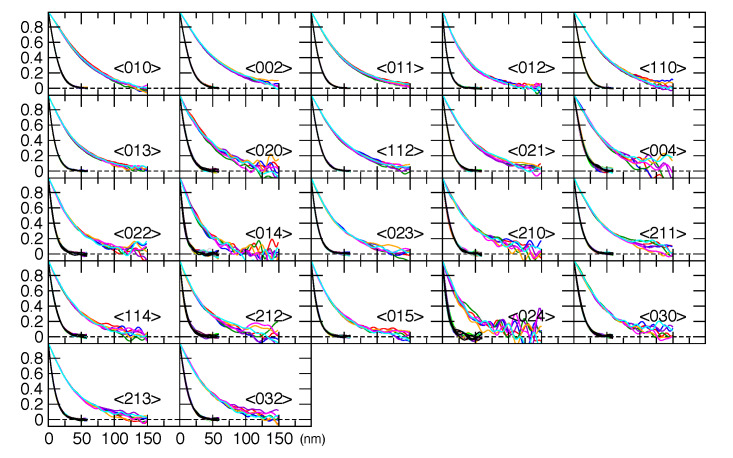
Fourier transforms of individual ZnO reflections from the NIST DBD.

*ℱ*$(f_{1})(k\omega_{0})=\frac{C\exp[-w\left|k\omega_{0}\right|-\frac{k^{2}\sigma^{2}\omega_{0}^{2}}{2}+A_{k}+iB_{k}}{ℱ\left(g_{IPF}\right)(k\omega_{0})}$ (19)

is the transform of the intrinsic peak shape from the material being analyzed. Note that, since *A_k_* and *B_k_* have uncertainties associated with them from the least-squares fit, these uncertainties can also be appropriately scaled and carried forward. The Fourier transforms resulting from this analysis are shown in Fig. 12 for the 11-BM machine and Fig. 13 for the NIST DBD. The horizontal scale of Fig. 12 extends to 200 nm with quite low noise, although there is no useful information about the sample above this value. The data from the DBD, Fig. 13 extends only to 150 nm, and the noise above this level negates any useful information.

**Fig. 14:**
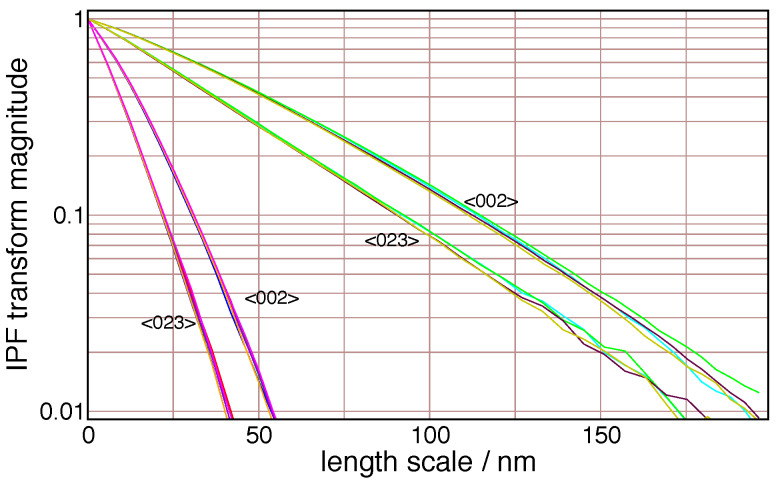
Detail of Fourier transforms of two ZnO reflections from the APS 11-BM data, on a log scale, illustrating the reduction in width in Fourier space caused by the Warren faulting model, which affects the <023> reflection but not the <002> reflection.

The Warren HCP stacking fault model introduces Lorentzian peak shape broadening; this leads to an exponential in the Fourier transform. Peaks that are heavily affected by stacking faults, such as the (023), fall off very rapidly in Fourier space (they are very wide in 2*θ* space). This effect is illustrated in Fig. 14. On a logarithmic scale, a pure exponential function is linear. The <002> reflection is unaffected by stacking faults, and it shows a strongly curved Fourier transform, which contains information that will be fit as a column length distribution. The <023> reflection is maximally broadened in real space by faulting; its transform is nearly linear, and falls off faster than that of the <002> reflection. Not much information about the column length distribution related to crystallite size can be derived from this, since the columns are all truncated by the stacking faults at a length scale much smaller than the crystallite size.

## 6 Determination of Certified Mean Column Lengths

The final step of the peak-by-peak analysis is the extraction of the area- and volume-weighted mean column lengths from these transforms. The area-weighted mean 〈*L*〉_area_ is the inverse of the derivative of the transform at the origin, per Eq. (12). The volume-weighted mean 〈*L*〉_vol_ is the integral of the transform from *ω* =-∞ to *ω* = ∞, per Eq. (14). These can be extracted by direct numerical integration and differentiation of the discrete transforms; this approach, however, has the disadvantage of being incapable of using the full statistical information derived from the underlying Poisson statistics of the data set. An alternative method that addresses this problem is to least-squares fit parametric functions to each transform, for which the derivatives and integrals can be computed analytically. We chose a log-normal size distribution model to parameterize the data for each peak. This model may not be physically correct in a rigorous manner, but it is known to be flexible enough to account for the expected level of deviation in transform shape, except in the case of a large degree of Lorentzian peak broadening. In this case, if the log-normal fitter fails to converge to meaningful values, the procedure falls back to the transform of a Voigt profile, which empirically is a very good model for high-angle peaks. The statistical properties of parameters computed from these fits correctly reflect the statistical properties of the underlying transforms. Although one could compute the formal Gaussian error ellipsoid from these values, in fact the statistical parameters computed for the certified results are determined from the sample replicates.

**Fig. 15:**
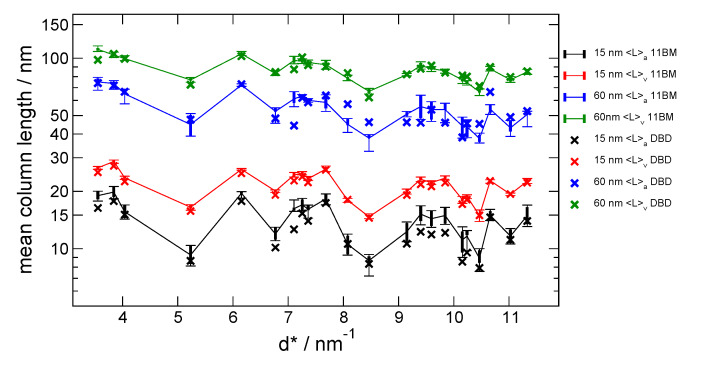
Results from Fourier analyses of the SRM 1979 data: certified values for 〈*L*〉_area_ and 〈*L*〉_vol_ from the 11-BM machine, and corresponding values from the DBD. Boxes are 25th percentile to 75th percentile of the samples; whiskers are extreme values.

Summary statistics of the certified 〈*L*〉_area_ and 〈*L*〉_vol_ values from the 11-BM data are shown in Fig. 15 as a function of the inverse *d*-spacing, d^*^, and the corresponding values from the DBD are also shown. The upper limit on d^*^ of 11.5 nm^-1^ shown in Fig. 15 is the highest value that could be measured using Cu K*α* radiation on the DBD. The 〈*L*〉_vol_ values for the 11-BM data are nearly independent of any aspect of the data analysis method because the resolution of machine is well in excess of that required for this measurement. The 〈*L*〉_vol_values for DBD agree reasonably well with the 11-BM values; the 15 nm values show a curious systematic bias in that the DBD values are nearly always slightly less than the values from 11-BM, and the 60 nm values simply show some degree of scatter throughout. This agreement is quite good given the limits in the resolution of the DBD shown in Fig. 10. The 〈*L*〉_area_ values are more sensitive to the accuracy in background subtraction, with its associated impact on the derivative of the Fourier transform at zero frequency. Hence, larger discrepancies are observed in the 〈*L*〉_area_ values for both size ranges between 11-BM and DBD machines. However the 11-BM data are certainly more accurate. Curiously, the same systematic bias is observed in the 15 nm values; any discrepancy between the DBD values and the 11-BM values is due to a reduced value for the DBD data. Again the 60 nm values simply show some degree of scatter throughout. The effect will be exacerbated for both weak and broadened reflections.

**Fig. 16:**
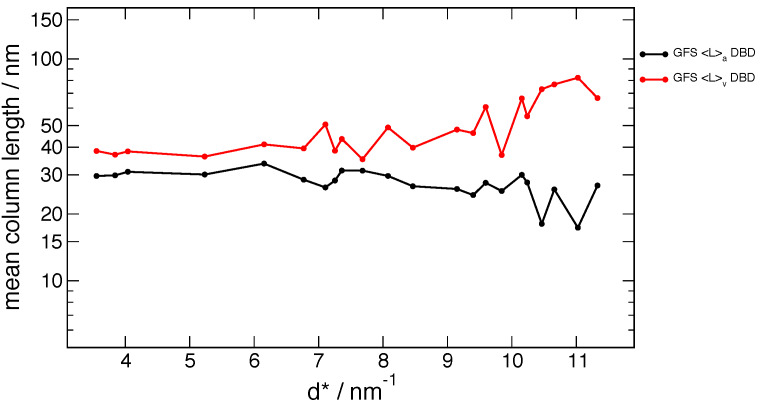
Moments measured for the ex-acetate flow-reacted ZnO material.

Figure 16 shows data from a single measurement of a sample of the ex-acetate ZnO prepared in the flow-reactor, which was not used in this project. The small ratio of 〈*L*〉_vol_ / 〈*L*〉_area_ ≈ 1.3 at low angles implies a relatively narrow width distribution with *σ* ≈ 0.4 [62]. However, the strong increase in 〈*L*〉_vol_ at higher d^*^ values is not easily explained by available models. The lack of *hkl* dependence following the Warren stacking fault model does imply that this material has a very low stacking fault density.

## 7 Fundamental Parameters Approach Refinements of ZnO Data

**Fig. 17:**
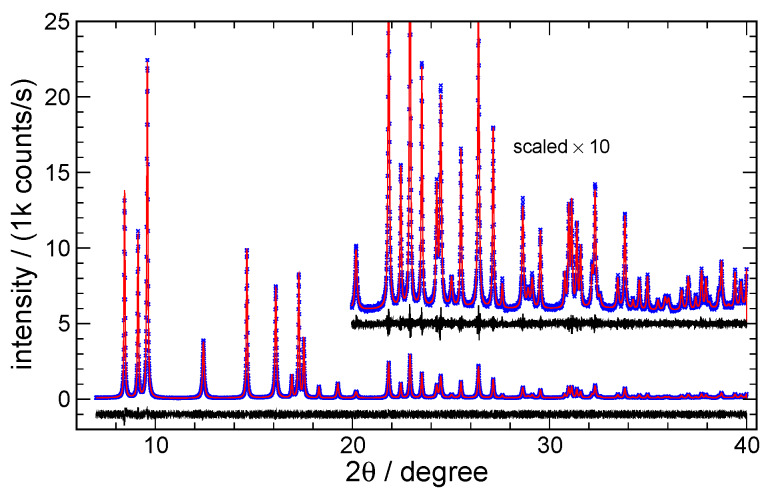

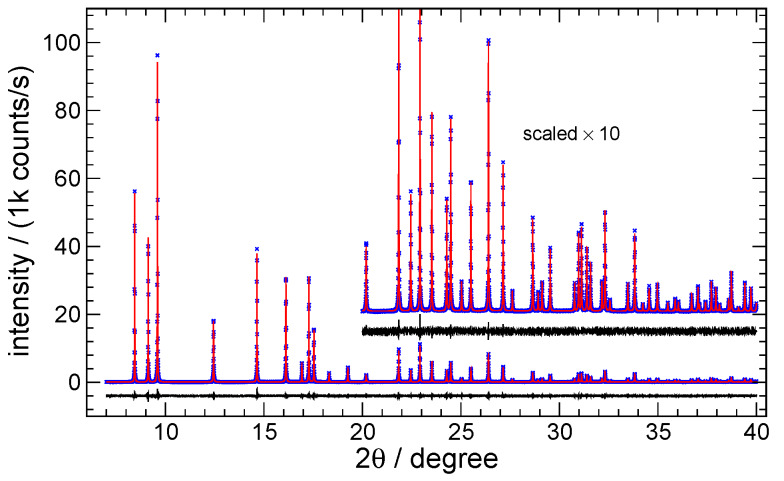
Typical FPA Pawley fits to ZnO data from 11-BM, using TOPAS, for (a) fit to 15 nm ZnO and (b) fit to 60 nm ZnO. Blue crosses indicate every 5th data point, red curves are the fit, and the black line is the residuals (offset for visibility).

**Fig. 18:**
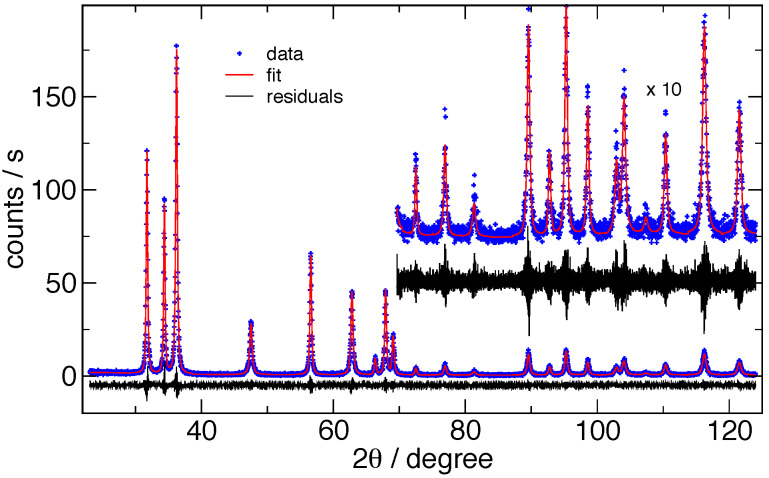

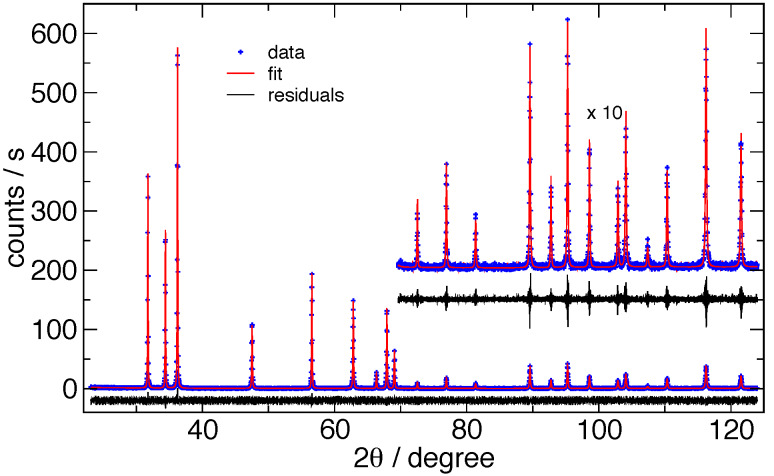
Typical FPA Pawley fits to ZnO data from the DBD, using FPAPC, for (a) fit to 15 nm ZnO and (b) fit to 60 nm ZnO. Blue crosses indicate every 5th data point, red curves are the fit, and the black line is the residuals (offset for visibility).

In order to consider the results from the Fourier methods with respect to more conventional approaches, we performed FPA analyses of the certification data sets using TOPAS and FPAPC. The IPF-specific parameters were fixed at those determined in Sec. 5.1. The crystallite size distribution was considered log-normal, with the model based on [38] for spherical crystallites; refined parameters consist of the median size, *D*_0_, and log-normal distribution width, *σ_l_*. The macro provided for using this model was corrected in house. These refinements were conducted as global fits, with multiple data sets being included in a single refinement. The Pawley fits included a six-term Chebyshev polynomial for the background as well as a 1*/*2θ ^2^ low angle tail. The stacking fault density model used was that of Warren. Lorentzian microstrain broadening, with width proportional to tan *θ*, was also included. Results from TOPAS and FPAPC were verified to be indistinguishable. Figure 17 shows the typical fits to 11-BM data, and Fig. 18 shows fits to the data from the DBD. Table 2 summarizes the parameters of interest from the 11-BM and DBD refinements, where these are reported as Information Values in the CoA. Improvements in the results from the 60 nm material with the use of the band-pass model are noteworthy and are illustrated in Ref. [60].

**Fig. 19:**
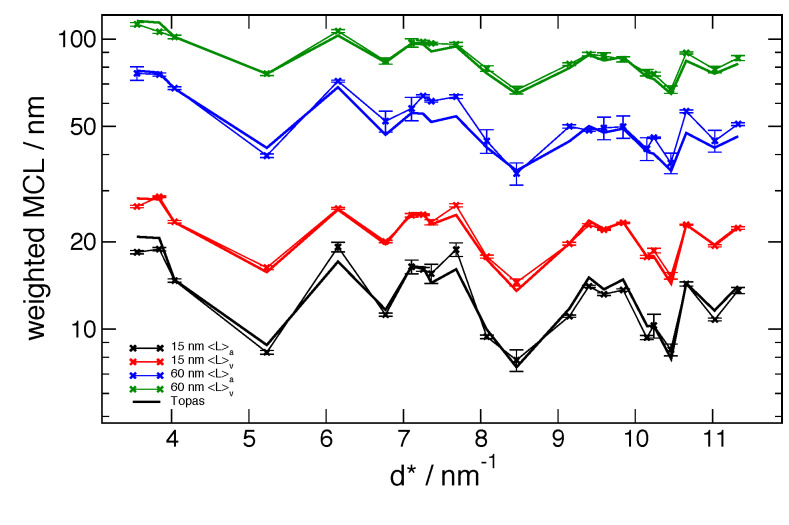
Certified peak-by-peak mean column lengths (MCL) from the 11-BM data vs. corresponding values from global FPA fits using parameters from Table 2. Error bars are Type A, statistical, *k* = 1 uncertainties of the mean.

**Fig. 20:**
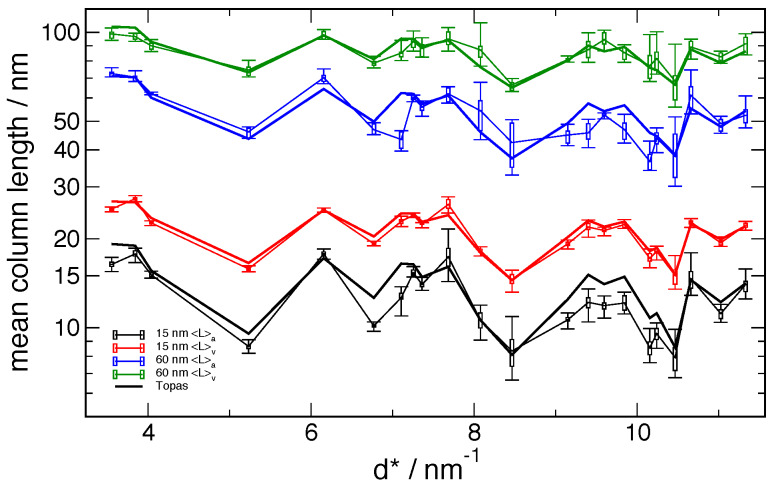
Values for Fourier, peak-by-peak mean column lengths, from the NIST DBD vs. corresponding values from global FPA fits using parameters from Table 2. Boxes are 25th percentile to 75th percentile of the samples; whiskers are extreme values.

**Fig. 21:**
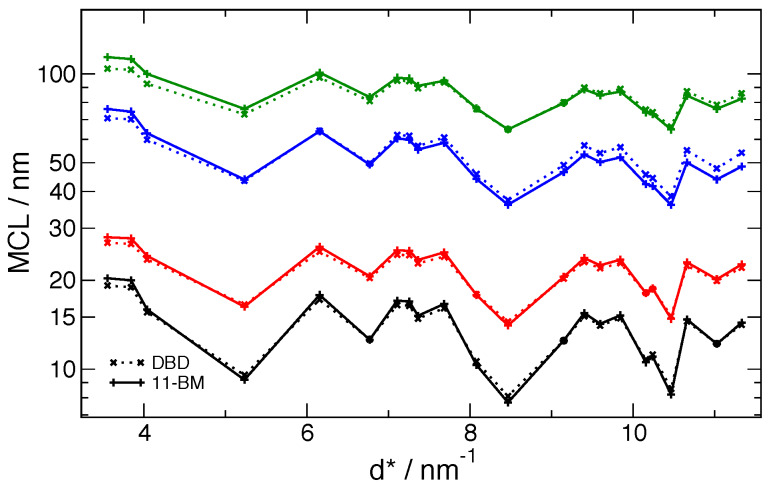
Comparison of 11-BM and DBD results from the FPA analyses. Average values of 〈*L*〉_area_ and 〈*L*〉_vol_ parameters are shown. Colors have same meaning as in Fig. 20.

The correspondence, or lack thereof, between the transform-based approach and the direct-fit approach can be investigated by synthesizing the ZnO peaks in Fourier space using FPAPC; see Sec. 5.1. The parameters shown in Table 2 are used as the input data for FPAPC, with the 〈*L*〉_area_ and 〈*L*〉_vol_ values being computed as aforementioned, Sec. 6. The values from the independent, Fourier analysis vs. FPA global fits are shown for the 11-BM machine in Fig. 19 and for the NIST DBD in Fig. 20. For the 11-BM data the Fourier results are, in fact, the certified values, and the reported error bounds are the Type A, statistical, *k* = 2 uncertainties of the mean, computed in accordance with those reported on the CoA. Results from the FPA analyses from the two machines are compared in Fig. 21.

**Table 2:** Information Values on the ZnO materials from TOPAS based refinements of the FPA using the spherical-crystallite log-normal size distribution model. The uncertainties are the esd values reported by TOPAS.

	15 nm, 11-BM	15 nm, DBD	60 nm, 11-BM	60 nm, DBD
〈L〉_area_(nm)	23.83(6)	23.77(30)	95.4(4)	80.7(13)
〈L〉_vol_ (nm)	31.39(9)	31.65(46)	138.9(6)	128.3(25)
median diameter *D*_0_ (nm)	24.11(4)	23.35(25)	75.0(1)	51.1(6)
distribution width *σ_l_*	0.397(1)	0.411(4)	0.508(1)	0.58(4)
deformation *α*	0.00121(2)	0.00159(10)	0.00028(1)	0.00035(2)
stacking fault *β*	0.01259(5)	0.01082(23)	0.00157(1)	0.00147(3)
strain *ε*_0_ / 10^-6^	200(2)	182(8)	73(1)	12(2)
*Χ*^2^*/N* (GoF)	1.11	1.06	1.04	1.09

An initial inspection of Fig. 19 and Fig. 20 indicates that, for both the 11-BM and DBD machines, excellent agreement is observed between the 〈*L*〉_vol_ values from the Fourier vs. FPA methods. In contrast to this, however, there are discrepancies between corresponding 〈*L*〉_area_ values, for both crystallite size ranges and both machines, although the results from 11-BM are observed to be superior to those from the DBD. These observations are essentially due to the same factors discussed with respect to Fig. 15. The 〈*L*〉_area_ values are obtained from a small number of transform values at the origin and are quite sensitive to errors in their values. The 〈*L*〉_vol_ values are determined from the integral of the transform and are thus relatively insensitive to errors in specific values. While the methods of Ref. [35] constitute a marked improvement in the determination of backgrounds level, what we are seeing is the last trace of error in the background determination that is known to strongly effect the 〈*L*〉_area_ values. Observing Fig. 21, an offset in MCL values is apparent between the two machines for both datasets and analysis methods. This is due to the fact that the DBD is in reflection geometry, and there is a small error in the FPA fitting of the IPF to low angle. While the issue is well known [63], the precise origin of it is not. Agreement is otherwise quite good, although 〈*L*〉_area_ values for the 60 nm material diverge with high angle. The origins of this behavior are not understood; however, we are certainly at the confidence limits of the models and refined parameters with these results.

**Fig. 22:**
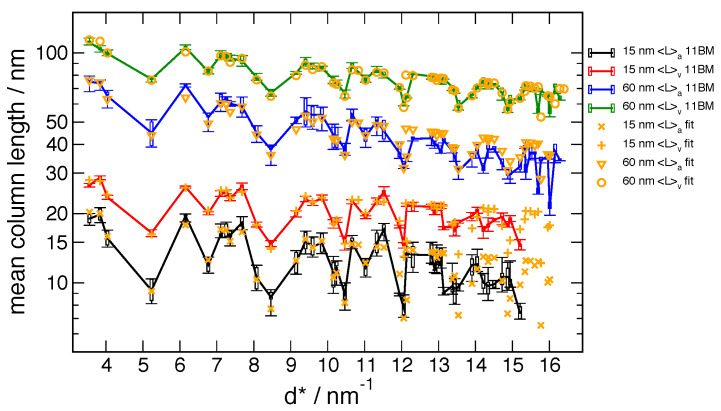
Peak-by-peak mean column lengths from 11-BM data, via both Fourier and FPA methods, displayed to extended values of d*.

The Warren stacking fault model, with its dependence on *hkl*, is shown to give results that match the strong *hkl*-dependent variation in the profile breadth. The quality of the match is emphasized by the correspondence of the 〈*L*〉_vol_ values of Fig. 19, wherein the other sources of error are known to be minimal. The gradual slope in the MCL downwards from left to right is the result of micro-strain. The limits to the microstructure models, even for 11-BM results, are highlighted in Fig. 22, which extends to higher d^*^ values than those included in the certification. It is evident that the 〈*L*〉_area_ values are significantly overestimated by the model relative to the measurement beyond d^*^ = 11 nm^-1^, and it appears that the Warren stacking fault dependence on *hkl* has broken down for 〈*L*〉_area_ values beyond d^*^ = 13 nm^-1^. The origins of these observations are not understood. Finally, the refined parameters of the log-normal crystallite size distribution, *D*_0_ and *σ_l_*, are highly correlated; they are capable of fitting the observation with quality, but differing sets of these refined parameters can generate nearly indistinguishable results in the context of the 〈*L*〉_area_ and 〈*L*〉_vol_ values.

**Fig. 23:**
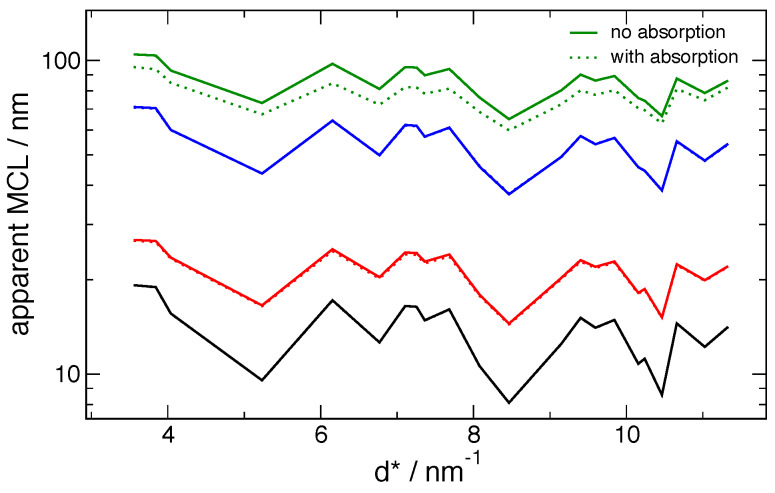
Attenuation correction for 〈*L*〉_vol_ and 〈*L*〉_area_ values as computed by FPAPC . Colors have same meaning as in Fig. 22.

With regards to LPA, specimen transparency is known to be an issue with data from divergent-beam, reflection geometry machines, such as the DBD. The refined attenuation corrections obtained for the DBD data used in the FPA analyses were observed to be non-physical. The value from the global fits of the 15 nm material was 85 cm^-1^, while that for the 60 nm material, with sharper lines was, 50 cm^-1^. Qualitative "tap density" measurements on the two powders indicated that they pack to ≈20% density and that the 15 nm material packed to about 90% of that for the 60 nm material. Given that the linear attenuation coefficient for zinc oxide is 277 cm^-1^, the figure of 50 cm^-1^ appears tenable. This is not surprising because the narrow lines of the 60 nm material would favor the proper functionality of the attenuation model; conversely, the broad lines of the 15 nm material would render is inoperable. The size of the absorption correction to the values of 〈*L*〉_area_ and 〈*L*〉_vol_ as computed with FPAPC is illustrated in Fig. 23. The 〈*L*〉_area_ values are unaffected as they are based on transform values at the origin, which are not subject to an attenuation correction. The 〈*L*〉_vol_ values of the 60 nm material are strongly affected; this is not unexpected because the sharp lines of this material are going to be significantly broadened by the reduction in attenuation of ZnO relative to the LaB_6_ of SRM 660b. Only a slight change in the 〈*L*〉_vol_ values for the 15 nm material is noted; the profiles for this material are quite broad by default. The capillary data from the 11-BM machine are not affected by variations in specimen transparency.

### 7.1 Lattice Parameter Measurement

Analysis of data from the NIST DBD provided lattice parameter values that are traceable to the SI owing to its accurately calibrated angular scale and its usage of the well-characterized Cu K*α* emission spectrum [59]. The FPA analyses were carried out as per methods delineated in Sec. 7. The sample attenuation issue was addressed with the use of zero background quartz plates for the specimen holders; no specimen attenuation correction was applied in the analyses of these data. Data from five such specimens were used to obtain the certified lattice parameters. The lattice constants were corrected to a standard temperature of 22.5° C using data from Ref. [64], page 444. The temperature coefficient of the lattice constant *a* can be computed to be 5.51 × 10^-6^ K^-1^ and that of *c* can be computed to be 3.29 × 10^-6^ K^-1^ at 300 K.

## 8 Statistical Analysis

The statistical analysis was performed in four parts: 1) The assessment of homogeneity with respect to lattice parameter, 2) The assessment of homogeneity with respect to parameters 〈*L*〉_area_ and 〈*L*〉_vol_, 3) Determination of Type A (statistical) and Type B (systematic) errors of the certified lattice parameters, and 4) Determination of Type A and Type B errors of the certified mean column lengths. Each of these independent efforts was carried out in two stages: exploratory/graphical and quantitative [65].

### 8.1 Homogeneity Assessment

The data analysis for the homogeneity verification was carried out on the data from the DBD. The lattice parameters were determined from TOPAS fits of the scintillation data refined as per Sec. 7 except that each data set was fit independently. The log-normal parameters for crystallite size distribution, median diameter and distribution width, analogous to the values shown in Table 2, were used as a proxy for the microstructural homogeneity of the ZnO material. These microstructural data were obtained using TOPAS to fit data sets collected on the DBD equipped with the PSD, again refined individually as per Sec. 7.

FPAPC was then used to generate the 22 values for 〈*L*〉_area_ and 〈*L*〉_vol_ using the refined parameters for each data-set. Statistical analysis of the lattice parameters and values for 〈*L*〉_area_ and 〈*L*〉_vol_, which included graphical block plots (see Sec. 1.3.3.3 in Ref. [65]) as well as Analysis of Variance, indicated that the SRM feedstock was homogeneous with respect to diffraction properties.

### 8.2 Certified Lattice Parameters

The certified lattice parameters are shown in Table 3. The type "A" errors are purely statistical, and are based on the *k* = 2 expanded standard error of the mean of the measurements. The type "B" errors are clearly dominant, and were estimated from instrumental and fitting systematics [60].

**Table 3:** Certified lattice parameters with expanded *k* = 2 Type A and Type A+B uncertainties.

	temperature	expanded (*k* = 2)	expanded (*k* = 2)
	corrected	Type A uncertainty	(A+B) uncertainty
	lattice parameter		
	(nm) 15 nm		
a c	0.3249766 0.5208376	±0.000 005 4 ±0.000 0194	±0.000 030 ±0.000 030
60 nm	
a c	0.3249872 0.5206804	±0:000 0080 ±0.000 0120	±0.000 020 ±0.000 020

### 8.3 Certified Mean Column Lengths

Four exploratory plots were generated for each set of five, or four, replicates: a run sequence plot, a lag-1 plot, a histogram, and a normal probability plot. Once these plots were observed to indicate satisfactory results, the quantitative calculations were carried out to compute the certified values. Owing to the straightforward nature of these analyses and the near-normality of the data, the sample mean was used as the certified value, and the *k* = 2 expanded standard error of the mean was computed as the Type A expanded uncertainty.

**Fig. 24:**
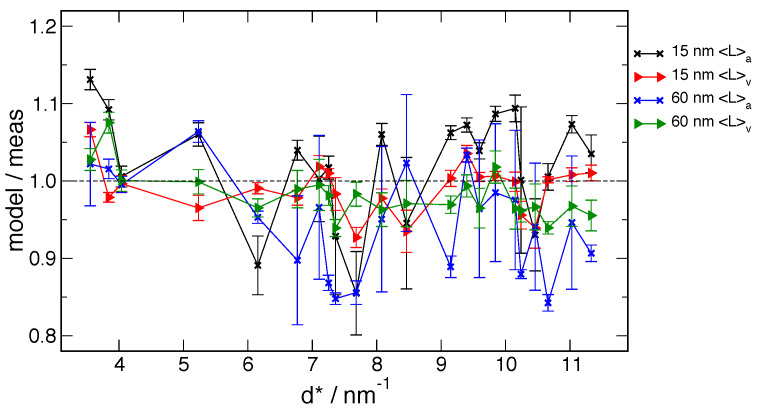
Peak-by-peak ratios of mean column lengths, as determined from the FPA refinements, relative to values from the Fourier analysis (certified values).

The Type B errors were assessed with a comparison of MCL values from the FPA fits with the certified values. The ratios of the 〈*L*〉_area_ and 〈*L*〉_vol_ values obtained from the FPA vs. those from the Fourier methods are illustrated in Fig. 24. One can see that the 〈*L*〉_vol_ ratios are consistently closer to unity than the 〈*L*〉_area_ ratios. The trends observed indicate that a 15% uncertainty is appropriate for the 〈*L*〉_area_ values, while the reasonable uncertainty for the 〈*L*〉_vol_ MCL values is 10%. The interval defined by the certified value and its uncertainty represents an expanded Type A + B uncertainty with *k* = 2, and it was calculated according to the method described in the ISO/JCGM Guide. The certified MCL values and statistics are shown in Tables 4 and 5.

**Table 4:** Certified peak shape parameters for the "15 nm" material with expanded *k* = 2 Type A and Type A+B uncertainties, with Information Value peak positions based on Cu K*α λ* = 0.154 059 29 nm.

2*θº*	*hkl*	〈*L*〉_area_ (nm)	(*k* = 2)	(*k* = 2)	〈*L*〉_vol_ (nm)	(*k* = 2)	(*k* = 2)
Type A				Type A+B	Type A		Type A+B
31.770	010	18.4	±0.22	±3.0	26.5	±0.22	±2.9
34.409	002	18.9	±0.22	±3.1	28.6	±0.16	±3.0
36.252	011	14.7	±0.18	±2.4	23.5	±0.22	±2.6
47.531	012	8.3	±0.11	±1.4	16.3	±0.24	±1.9
56.598	110	19.2	±0.65	±3.5	26.1	±0.17	±2.8
62.840	013	11.2	±0.13	±1.8	20.1	±0.17	±2.2
66.379	020	16.4	±0.81	±3.3	24.7	±0.31	±2.8
67.942	112	16.1	±0.21	±2.6	24.8	±0.13	±2.6
69.088	021	15.5	±1.05	±3.4	23.4	±0.44	±2.8
72.536	004	18.8	±0.91	±3.7	26.7	±0.31	±3.0
76.955	022	9.4	±0.12	±1.5	17.8	±0.19	±2.0
81.357	014	7.8	±0.59	±1.8	14.5	±0.35	±1.8
89.599	023	11.1	±0.09	±1.8	19.7	±0.18	±2.2
92.798	210	14.1	±0.11	±2.2	22.9	±0.21	±2.5
95.310	211	13.2	±0.12	±2.1	22.0	±0.11	±2.3
98.591	114	13.6	±0.12	±2.2	23.3	±0.12	±2.5
102.931	212	9.4	±0.14	±1.6	17.8	±0.20	±2.0
104.088	015	10.3	±0.87	±2.4	18.7	±0.30	±2.2
107.410	024	8.5	±0.35	±1.6	15.3	±0.36	±1.9
110.394	030	14.3	±0.22	±2.4	22.9	±0.08	±2.4
116.263	213	10.8	±0.11	±1.7	19.4	±0.15	±2.1
121.563	032	13.6	±0.30	±2.3	22.3	±0.20	±2.4

**Table 5:** Certified peak shape parameters for the "60 nm" material with expanded *k* = 2 Type A and Type A+B uncertainties, with Information Value peak positions based on Cu K*α λ* = 0.154 059 29 nm.

2*θ*ͦ	*hkl*	〈*L*〉_area_ (nm)	(*k* = 2)	(*k* = 2)	〈*L*〉_vol_ (nm)	(*k* = 2)	(*k* = 2)
Type A				Type A+B	Type A		Type A+B
31.766	010	76.1	±4.11	±15.5	112.4	±1.59	±12.8
34.419	002	75.5	±0.95	±12.3	106.0	±1.42	±12.0
36.251	011	67.7	±0.72	±10.9	101.7	±1.40	±11.6
47.535	012	39.6	±0.55	±6.5	76.0	±1.19	±8.8
56.591	110	71.6	±0.56	±11.3	106.5	±1.26	±11.9
62.852	013	52.1	±4.34	±12.2	84.0	±2.03	±10.4
66.371	020	57.6	±5.35	±14.0	97.0	±3.22	±12.9
67.942	112	63.7	±0.62	±10.2	97.7	±1.25	±11.0
69.081	021	61.1	±0.45	±9.6	96.6	±1.06	±10.7
72.559	004	63.3	±0.96	±10.5	95.9	±1.49	±11.1
76.953	022	44.5	±4.19	±10.9	79.1	±1.72	±9.6
81.377	014	34.4	±3.04	±8.2	66.7	±2.00	±8.7
89.604	023	50.0	±0.69	±8.2	82.0	±0.92	±9.1
92.784	210	48.5	±0.44	±7.7	88.7	±1.28	±10.2
95.298	211	49.4	±4.39	±11.8	87.4	±2.24	±11.0
98.608	114	49.9	±4.44	±11.9	85.2	±1.77	±10.3
102.923	212	41.9	±3.78	±10.1	76.6	±2.03	±9.7
104.122	015	45.8	±0.26	±7.1	75.8	±1.12	±8.7
107.425	024	37.4	±3.07	±8.7	67.0	±1.99	±8.7
110.375	030	56.3	±0.60	±9.0	89.6	±0.72	±9.7
116.262	213	44.6	±3.84	±10.5	78.5	±2.05	±9.9
121.549	032	50.9	±0.54	±8.2	86.0	±1.71	±10.3

## 9 Conclusions

The area-weighted mean column lengths, 〈*L*〉_area_, and the volume-weighted mean column lengths, 〈*L*〉_vol_, of the sample contribution to the breadth of each reflection, which were derived directly from the Fourier transform of the individual peak shapes, are the primary quantities that were certified for this SRM. These values are fundamental properties of the material being certified and essentially free of model-dependent interpretation. The 11-BM instrument selected for use in collecting the certification data has been demonstrated to be operating in conjunction with theoretical expectations in the *d*-space range of interest and imparting a minimum of influence on the certified parameters. These quantities are reported with an assessment of both the Type A (statistical) and Type B (systematic) measurement uncertainties. The lattice parameters are also certified. The crystallite size distributions, determined from an FPA analysis on the data from the two machines, are provided as Information Values since there is model-dependent interpretation involved in such an analysis. The ZnO materials of this SRM present a wide range of peak widths that are affected by various well-understood physical characteristics. This makes this material a particularly good candidate for a line shape standard in that it offers not only complex measurement issue; but that, through the course of the certification, we have quantitatively addressed contributions to the profile breadths of this SRM. Data from the NIST machine equipped with the IBM, when analyzed with use of the "band-pass" model yielded credible results from the 60 nm material; this size range was considered to be at or near the upper limit of the measurement capability of laboratory equipment.

Supplemental Materials

• Supplemental files DOI: https://doi.org/10.18434/M32269

• Diffraction patterns collected in support of this work are available as CIF files at the DOI above.
